# The phycoerythrobilin isomerization activity of MpeV in *Synechococcus* sp. WH8020 is prevented by the presence of a histidine at position 141 within its phycoerythrin-I β-subunit substrate

**DOI:** 10.3389/fmicb.2022.1011189

**Published:** 2022-11-15

**Authors:** Lyndsay A. Carrigee, Jacob P. Frick, Xindi Liu, Jonathan A. Karty, Jonathan C. Trinidad, Irin P. Tom, Xiaojing Yang, Louison Dufour, Frédéric Partensky, Wendy M. Schluchter

**Affiliations:** ^1^Department of Biological Sciences, University of New Orleans, New Orleans, LA, United States; ^2^Environmental Laboratory, Engineering and Research Development Center, US Army Corps of Engineers, Vicksburg, MS, United States; ^3^Department of Chemistry, Indiana University, Bloomington, IN, United States; ^4^Department of Chemistry, University of Illinois Chicago, Chicago, IL, United States; ^5^Ecology of Marine Plankton Team, UMR 7144 Adaptation and Diversity in the Marine Environment, Station Biologique, Sorbonne Université, CNRS, Roscoff, France

**Keywords:** bilin lyase, cyanobacteria, chromatic acclimation, phycobilisome, phycoerythrin, phycoerythrobilin, phycourobilin, post-translational modification

## Abstract

Marine *Synechococcus* efficiently harvest available light for photosynthesis using complex antenna systems, called phycobilisomes, composed of an allophycocyanin core surrounded by rods, which in the open ocean are always constituted of phycocyanin and two phycoerythrin (PE) types: PEI and PEII. These cyanobacteria display a wide pigment diversity primarily resulting from differences in the ratio of the two chromophores bound to PEs, the green-light absorbing phycoerythrobilin and the blue-light absorbing phycourobilin. Prior to phycobiliprotein assembly, bilin lyases post-translationally catalyze the ligation of phycoerythrobilin to conserved cysteine residues on α- or β-subunits, whereas the closely related lyase-isomerases isomerize phycoerythrobilin to phycourobilin during the attachment reaction. MpeV was recently shown in *Synechococcus* sp. RS9916 to be a lyase-isomerase which doubly links phycourobilin to two cysteine residues (C50 and C61; hereafter C50, 61) on the β-subunit of both PEI and PEII. Here we show that *Synechococcus* sp. WH8020, which belongs to the same pigment type as RS9916, contains MpeV that demonstrates lyase-isomerase activity on the PEII β-subunit but only lyase activity on the PEI β-subunit. We also demonstrate that occurrence of a histidine at position 141 of the PEI β-subunit from WH8020, instead of a leucine in its counterpart from RS9916, prevents the isomerization activity by WH8020 MpeV, showing for the first time that both the substrate and the enzyme play a role in the isomerization reaction. We propose a structural-based mechanism for the role of H141 in blocking isomerization. More generally, the knowledge of the amino acid present at position 141 of the β-subunits may be used to predict which phycobilin is bound at C50, 61 of both PEI and PEII from marine *Synechococcus* strains.

## Introduction

**Figure 1:**
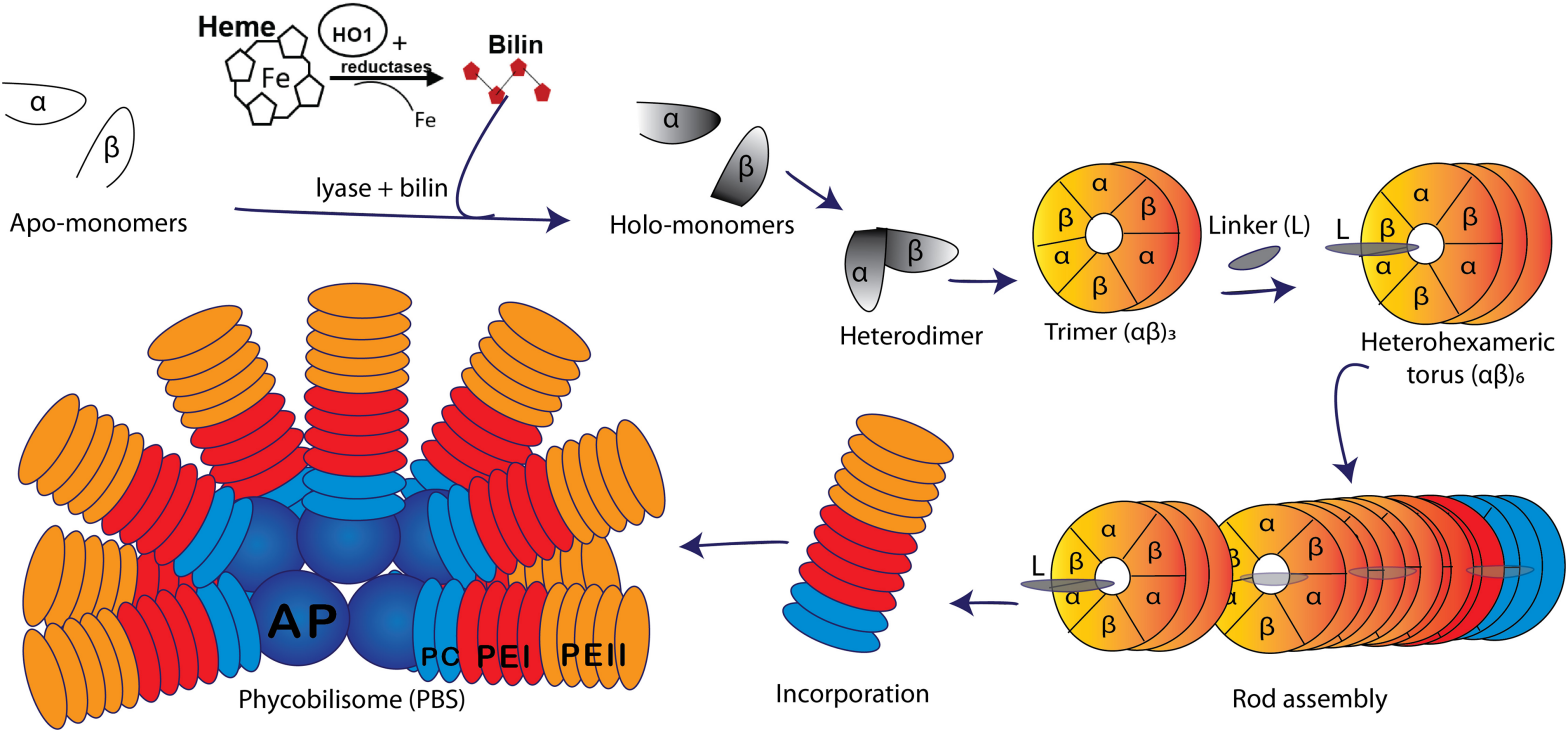
Model of a marine *Synechococcus* WH8020 phycobilisome (PBS) containing phycoerythrin I (PEI, red), phycoerythrin II (PEII, orange), phycocyanin (PC, light blue), and an allophycocyanin (AP, deep blue) core. A depiction of PBS rod assembly shows the addition of bilin *via* lyase catalyzed post-translational modification of apo-α and apo-β monomers (white) forming holo-monomers (gray) which come together to form a heterodimer. Heterodimers are subsequently arranged in trimers (αβ)_3_ followed by heterohexamers (αβ)_6_ and with the help of linker (L) polypeptides, the PBS rod is formed and bound onto the core (Glazer, 1982; Glazer, 1984; Six et al., 2007; Schluchter et al., 2010).

Marine cyanobacteria are responsible for as much as half of the world’s oxygen production and photosynthesis and play a key role in carbon and nutrient cycling (Bengston, 1994; Kasting and Siefert, 2003; Kehoe, 2010). Marine isolates of *Synechococcus* cyanobacteria possess huge light-harvesting complexes (or phycobilisome; hereafter PBS), comprised of up to four types of highly pigmented phycobiliproteins (PBPs). Allophycocyanin constitutes the core of the PBS, surrounded by 6–8 rods made of phycocyanin and up to two types of phycoerythrin (PEI and PEII; Figure 1; Ong and Glazer, 1991; Glazer, 1994; Everroad et al., 2006; Flombaum et al., 2013; Sanfilippo et al., 2019a). This variable PBP content extends the spectral range of the PBS light harvesting capabilities. PEI and PEII are homologous PBP, each composed of an α- and a β-subunit arranged in a torus-like hetero-hexamer (αβ)_6_ and stacked together with the help of linker polypeptides to form the distal portion of the PBS rods (Figure 1; Glazer, 1988; Betz, 1997; Schluchter et al., 2010).

The large pigment diversity in marine strains of *Synechococcus* PBS is not only due to its variable PBP content but also to the variable composition of covalently bound linear tetrapyrrole bilins. The latter are post-translationally added to highly conserved cysteine (C) residues of PBP precursors by a variety of bilin lyases. Three major groups or clans of bilin lyases have been characterized to date: the S/U type, the T type, and the E/F type (Fairchild et al., 1992; Fairchild and Glazer, 1994; Shen et al., 2006; Zhao et al., 2007; Saunée et al., 2008). Bilin chromophore and attachment site specificity as well as primary amino acid sequence similarities are trademarks differentiating members of each clan. The crystal structures of the S/U [Protein Data Bank, (PDB): 3BDR; Zhao et al., 2006; Shen et al., 2008;Schluchter et al., 2010; Kronfel et al., 2013; Overkamp et al., 2014] and T (PDB: 4O4O; Zhou et al., 2014; Gasper et al., 2017) lyases show that they adopt a similar antiparallel beta-barrel structure akin to the lipocalin protein family (Schluchter et al., 2010; Kronfel et al., 2013; Overkamp et al., 2014). The E/F lyases adopt all helical structures. While the crystal structure of the heterodimeric CpcE/F lyase was reported to adopt an α-helical structure of a solenoid shape (Zhao et al., 2017), the recent crystal structure of a novel lyase-isomerase, MpeQ (Kumarapperuma et al., 2022), suggested that a question mark-like architecture represents a common protein framework for both single chain and heterodimeric E/F lyases (Andrade et al., 2001; Marcotrigiano et al., 2001; Kozo et al., 2002; Takano and Gusella, 2002; Scheer and Zhao, 2008; Schluchter et al., 2010; Zhao et al., 2017; Kumarapperuma et al., 2022). The S/U lyases exhibit a broad variation in chromophore and PBP substrate specificity but are highly specific with regard to C binding sites (Shen et al., 2006; Saunée et al., 2008; Scheer and Zhao, 2008; Biswas et al., 2010; Schluchter et al., 2010; Kronfel et al., 2013; Zhou et al., 2014; Gasper et al., 2017). In contrast, the E/F lyases display high specificities for the bilin chromophore and bilin binding site on a particular PBP (PDB 5N3U; Fairchild et al., 1992; Swanson et al., 1992; Zhou et al., 1992; Fairchild and Glazer, 1994).

**Figure 2:**
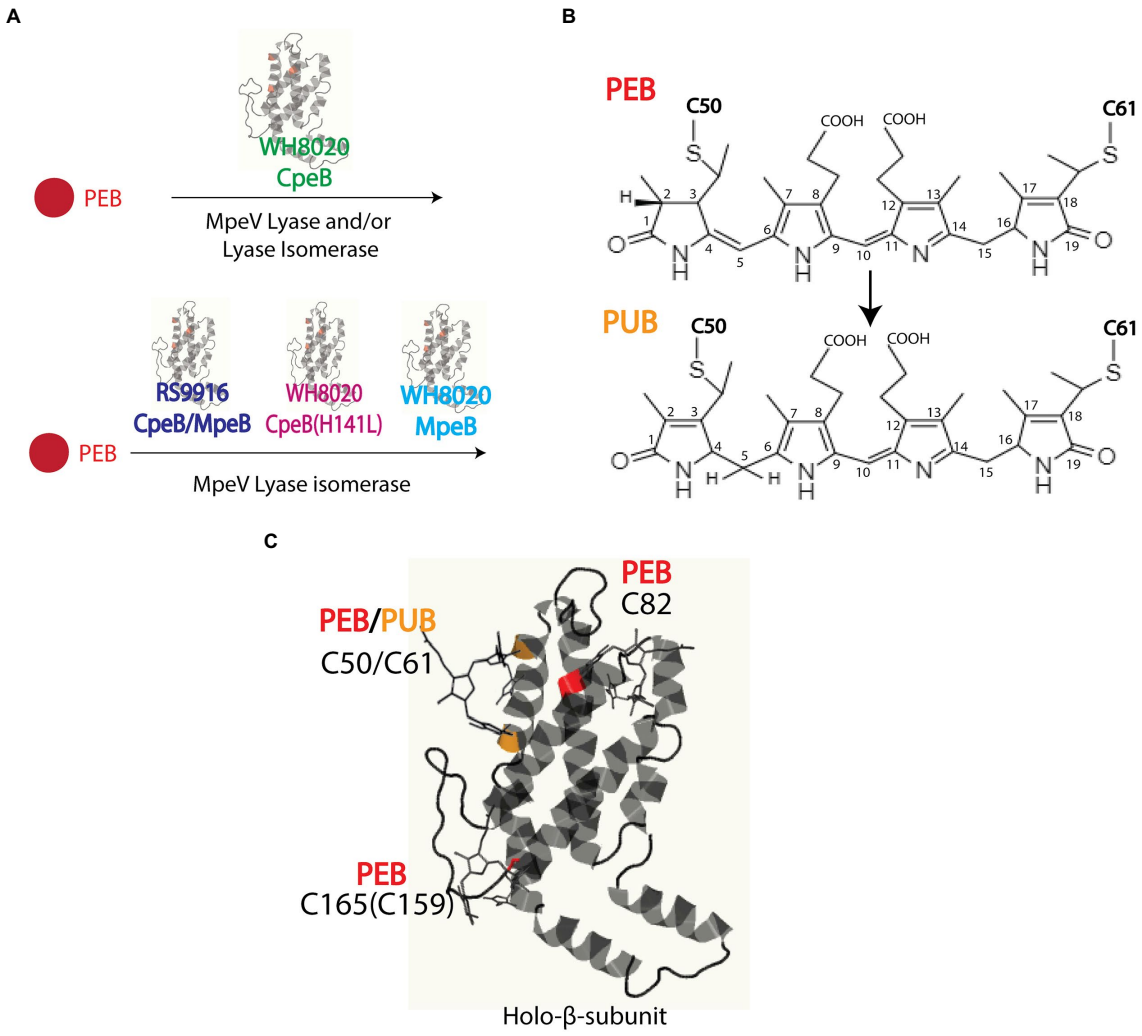
**(A)** Model of *Synechococcus* sp. WH8020 MpeV activity as both a lyase and lyase-isomerase. **(B)** Chemical structures of phycoerythrobilin (PEB) and doubly-linked phycourobilin (PUB). Post-translational pigment attachment is catalyzed by bilin lyases or lyase isomerases with a thioether linkage at the 3^1^ carbon of the bilin A ring during single attachment or additionally through the 18^1^ carbon of the bilin D ring when doubly attached. **(C)** Bilin structure and ligation sites, representative of RS9916 and WH8020 CpeB. Ribbon diagram generated using Phyre^2^ (Kelley et al., 2015). depicting β-subunit of PEI (β-PEI) chromophorylation pattern and lyases involved (bilins in ball and stick). PEB ligated residues are highlighted in red, and PUB ligated residues are highlighted in orange.

Marine strains of *Synechococcus* containing both PEI and PEII possess a diverse set of E/F lyases (Wilbanks and Glazer, 1993; Six et al., 2007; Shukla et al., 2012), particularly in those strains that undergo Type IV chromatic acclimation (CA4). In the CA4 phenomenon, *Synechococcus* are able to alter the ratio between the blue light-absorbing chromophore phycourobilin (PUB; λ_max_ ~ 495 nm) and the green light-absorbing chromophore phycoerythrobilin (PEB; λ_max_ ~ 545 nm) in both PEI and PEII, thereby extending the light harvesting capabilities of PBS (Figures 1, 2; Palenik, 2001; Everroad et al., 2006; Kehoe, 2010; Humily et al., 2013; Sanfilippo et al., 2016, 2019a,b). This unique CA4 phenomenon is conferred by a small genomic island that occurs in two possible configurations, CA4-A or CA4-B, (Supplementary Figure 1; Humily et al., 2013). Each island encodes a transcriptional activator, a repressor, a protein of unknown function, and an E/F-type bilin lyase (PEB lyase MpeW in the CA4-B island) or lyase-isomerase (MpeZ in the CA4-A island; Supplementary Figure 1). A bilin lyase-isomerase is an enzyme that has an additional activity to isomerize PEB to PUB during the ligation reaction (Everroad et al., 2006; Shukla et al., 2012; Humily et al., 2013; Sanfilippo et al., 2016).

Intriguingly, all marine *Synechococcus* containing a CA4-A island also possess a specific member of the E/F clan, MpeV, which is not involved in the CA4 process (Carrigee et al., 2020b). The *mpeV* gene is located in a large genomic region containing a cluster of genes involved on the biosynthesis of PBS rods, so-called the “PBS genomic region” (Supplementary Figure 1; Wilbanks and Glazer, 1993; Grébert et al., 2021). We recently characterized MpeV from the model *Synechococcus* CA4-A strain RS9916 (hereafter RS9916). In RS9916, we showed that MpeV is integral in the ligation reaction of a doubly-linked PUB at the equivalent position (C50, 61) on the recombinant β-subunits of both PEI (CpeB) and PEII (MpeB), demonstrating that MpeV is a lyase-isomerase capable of attaching the doubly-linked PUB on both subunits (see Figure 2; Carrigee et al., 2020b). This study also showed that RS9916 MpeV activity requires ligation of PEB at C82 position on both CpeB and MpeB by CpeS (a S/U type lyase) and is enhanced by activity of the chaperone-like enzyme CpeZ (a member of the E/F clan; Kronfel et al., 2019a,b; Carrigee et al., 2020b). When all three enzymes (CpeS, CpeZ, and MpeV) from RS9916 were expressed, both CpeB and MpeB possessed a doubly-linked PUB at C50, 61 (Carrigee et al., 2020b).

It has been reported that in *Synechococcus* sp. WH8020 (hereafter WH8020) PEI and PEII, CpeB incorporates PEB while MpeB has PUB at the C50, 61 positions (Ong and Glazer, 1991). Like RS9916, WH8020 is also a CA4-A strain that possesses *mpeV* (Wilbanks and Glazer, 1993; Carrigee et al., 2020b; Grébert et al., 2022). To determine whether MpeV is responsible for ligating PEB on CpeB and PUB on MpeB in WH8020, we employed recombinant protein expression, absorbance and fluorescence spectroscopy, and tandem mass spectrometry. We found that a single amino acid substitution on CpeB, specifically a change from leucine (L) to histidine (H) at position 141, was sufficient to block the isomerase activity of WH8020 MpeV. We further predict that a few other CA4-A strains containing a CpeB with the same substitution might have a similar bilin pattern, and, more generally, that the knowledge of the amino acid present at position 141 of CpeB or MpeB can be used to predict which phycobilin is bound at C50, 61 of both β-subunits from marine *Synechococcus* strains at large. We also propose a structural-based mechanism for the role of H141 in blocking isomerization.

## Materials and methods

### Plasmids for the characterization of WH8020 MpeV and CpeB

The putative lyase genes *cpeS, cpeZ,* and *mpeV* from the RS9916 genome (Supplementary Figure 1) were amplified *via* polymerase chain reaction (PCR) using a standard *Pfu* DNA polymerase system (ThermoFisher Scientific, Waltham, MA) and synthetic forward and reverse oligonucleotide primers with engineered restriction endonuclease sites (Eurofins MWG Operon, Huntsville, AL). Primers used to amplify genes by PCR for the construction of these expression vectors are previously published (Carrigee, 2020; Carrigee et al., 2020a,b). Amplified fragments were separately cloned into compatible Novagen Duet vectors using corresponding restriction enzymes as listed in Supplementary Tables 1, 2 or were previously published (Carrigee, 2020; Carrigee et al., 2020a,b). Expression vectors used in this study (Supplementary Table 2) include two previously described (Shukla et al., 2012; Kronfel et al., 2019b).

The *mpeV* gene from WH8020 was amplified *via* PCR using Platinum SuperFi II DNA Polymerase as a master mix in lieu of the *Pfu* system as previously described (Carrigee et al., 2020b). PCR reactions were performed using the standard High Fidelity PCR protocol from ThermoFisher Scientific (Waltham, MA). WH8020 substrate genes were cloned as an operon for PEI (*cpeBA*) with the beta subunit in frame with a hexahistidine tag (HT) and inserted into pET-DUET independently. WH8020 *mpeV* was cloned in-frame with the coding region of the HT and inserted into pCDF-DUET1. For co-expression of multiple genes required to characterize MpeV, some genes were subcloned into both multiple cloning sites (MCSs) for compatibility as follows. RS9916 *cpeZ* was cloned using Platinum SuperFi PCR protocol and inserted in frame after the sequence encoding a HT into MCSI of pCOLA-Duet containing non-tagged (NT) *cpeS* in MCSII (Carrigee, 2020; Carrigee et al., 2020b). The RS9916 *cpeA* gene sequence was inserted into multiple cloning site I (MCSI) pET-Duet (Novagen, Madison, WI) in frame with the sequence encoding a hexahistidine tag (HT). *cpeB* was subsequently subcloned into MCSII to achieve (MCSI/MCSII/vector) RS9916 HT*cpeA*/HT*cpeB*/pET-DUET and RS9916 HT*mpeA*/HT*mpeB*/pET-DUET as previously described (Carrigee, 2020; Carrigee et al., 2020b).

A single-site variant of WH8020 CpeB was created by mutating the H141 residue to L (H141L) using combined overlap extension PCR method adapted from (Hussain and Chong, 2016) using the Platinum SuperFi protocol (Supplementary Table 1). A PCR fragment containing the entire mutant WH8020 *cpeBA* operon was cloned into MCSI of pET-DUET vector containing resulting plasmids listed in Supplementary Table 2. All plasmids were sequenced for verification of clones and mutations (Eurofins Genomics LLC, Louisville, KY).

### *Escherichia coli* growth conditions and recombinant expression

Initial experiments for heterologous protein expression were performed using *E. coli* grown in Luria Bertani (LB) medium. However, we used modified, auto-induced medium for maximal protein yield (Studier, 2005). This involved a 100-ml LB starter culture of *E. coli* cells grown at 37°C overnight; then this was added to a liter of auto-induced medium composed of LB containing 1 mM MgSO_4_, 25 mM (NH_4_)_2_SO_4_, 50 mM KH_2_PO_4,_ 50 mM Na_2_HPO_4_, 0.5% glycerol, and 0.05% glucose at 18°C with appropriate combinations of antibiotics ampicillin (Ap: 100 μg·ml^−1^), chloramphenicol (Cm: 34 μg·ml^−1^), kanamycin (Km: 50 μg·ml^−1^), and spectinomycin (Sp: 100 μg·ml^−1^). With either medium, once the OD_600nm_ reached 0.6, cultures were induced with 1 mM isopropyl 1-β-D-thiogalactopyranoside, after which the cells were allowed to grow at 18°C for an additional 24 h before being harvested by centrifugation at 11,000 ×*g* for 8 min in a Sorvall RC 5C Plus centrifuge (Kendro Laboratory Products, Newtown, CT). Cell pellets were stored at −20°C until ready for purification and analysis. The wet weight of all cell pellets was measured and recorded prior to storage at −20°C.

### Protein purification

The histidine-tagged (HT) proteins were purified as previously described (Carrigee et al., 2020b). Briefly, cell pellets were resuspended at 3.0 ml·g^−1^ complete with mini protease cocktail (Thermo Scientific, Waltham, MA), 0.01 mg·ml^−1^ lysozyme (Fisher Scientific, Hampton, NH), and passed through a French Pressure Cell Press at 18,000 psi three times. All samples purified by cobalt affinity chromatography, dialyzed to remove imidazole, and then concentrated by ultrafiltration through an Amicon Ultra centrifugal filter unit (10 kDa cutoff; Novagen/EMD Millipore Corp., Darmstadt, Germany) and stored at −20°C.

### Analysis of recombinant protein and bound bilin

Purified protein was quantified using Bradford colorimetric assay (BioRad, Hercules, CA) and diluted to obtain equal concentrations across co-expressions for direct comparison when possible. Absorbance spectroscopy was performed using Perkin Elmer Lambda 35 UV/VIS or Shimadzu UV-2600 UV–Vis spectrophotometers followed by fluorescence spectroscopy using a Perkin Elmer LS55 (Waltham, MA) with excitation at 490 nm (PEB) or 440 nm (PUB; slit widths were set at 10 nm). Proteins were subsequently resolved by 15% (w/v) polyacrylamide gel electrophoresis (PAGE) in the presence of sodium dodecyl sulfate (SDS) and ultimately visualized by Coomassie blue staining (Saunée et al., 2008). To visualize proteins with bound bilin, gels were subjected to zinc-enhanced fluorescence using ChemiDoc MP imaging system (Bio-Rad, Hercules, CA) with excitation at 460–490 nm (PUB) and 520–545 nm (PEB).

### Growth of cyanobacterial strains

WH8020 cells were obtained from the Roscoff Culture collection.^1^ Cultures of WH8020 were grown at 22°C in PCR-S11 media and acclimated for at least 7 days in either blue light (BL) or green light (GL) and PBS were collected as previously described (Sanfilippo et al., 2016; Mahmoud et al., 2017; Sanfilippo et al., 2019b). Fluorescence excitation spectra were recorded at an emission of 575 nm, using a Perkin Elmer LS-50B spectrofluorometer. The fluorescence excitation 495–545 nm ratio was calculated and used as a proxy for the molar PUB to PEB ratio, as described (Humily et al., 2013).

### HPLC separation of PBS, trypsin digestion, and liquid chromatography tandem mass spectrometry

PBS were purified using methods previously described (Shukla et al., 2012; Sanfilippo et al., 2016, 2019a). Samples were dialyzed overnight against 5 mM Na phosphate buffer (pH 7.0) and subsequently purified *via* high performance liquid chromatography (HPLC) using methods outlined in (Shukla et al., 2012; Sanfilippo et al., 2016). Phycobiliproteins were monitored from 210 to 700 nm with specific channels monitoring for total protein (280 nm), PUB (490 nm), and PEB (550 nm). Relevant fractions were vacuum-dried and kept at −20°C prior to digestion for mass spectrometric (MS) analysis as previously described (Biswas et al., 2011; Shukla et al., 2012; Sanfilippo et al., 2016). Purified proteins were dialyzed against 2 mM sodium phosphate buffer (pH 7.0) and 1 mM β-mercaptoethanol. One aliquot of trypsin (dimethylated trypsin from porcine pancreas; Sigma, St. Louis, MO) was added to 2% (w/w) from a 20 μg ml^−1^ stock to the denatured protein mixtures and incubated at 30°C for 3 h in the dark (Shukla et al., 2012). The reaction was quenched by adding 30% (v/v) glacial acetic acid. Digested peptides were passed through a pre-equilibrated C8 Sep-Pak cartridge (Waters Corporation, Milford, MA), and thereafter the eluted sample was vacuum dried and stored at −80°C before LC–MS^2^ on a Thermo Orbitrap Fusion Lumos instrument. MS1 scans were obtained with a resolution of 120,000 and mass range of 300–2,000 m/z. Data dependent HCD were acquired with a 3 s cycle time, quadrupole precursor isolation window of two 2 m/z and resolution of 30,000 with 30% relative collision energy. Samples were separated by an Easy NanoLC1200 HPLC (ThermoFisher) equipped with a 75 μm × 15 cm Acclaim PepMap100 separating column (Thermo Scientific) downstream of a 2 cm guard column (Thermo Scientific). Buffer A was 0.1% formic acid in water. Buffer B was 0.1% formic acid in 80% acetonitrile. Peptides were separated on a 30 min gradient from 4% B to 33% B. All data processing was performed with Thermo XCalibur 4.0, Proteome Discover 2.1 (Thermo Scientific), and a local copy of ProteinPropector 5.22.1 (prospector.ucsf.edu).

### Bioinformatics and structural modeling of proteins

Gene sequences from *Synechococcus* strains RS9916 and WH8020 were retrieved from Cyanorak (Garczarek et al., 2021). Amino acid sequences were analyzed using the ClustalW alignment tool from MacVector software V. 12.7.5 (MacVector Inc., Apex, NC) and Phyre^2^ prediction system (Kelley et al., 2015). The model of the MpeV-CpeB complex was obtained using the AlphaFold2 multimer implemented in the ColabFold server based on the protein sequences of MpeV and CpeB from RS9916 (Jumper et al., 2021; Mirdita et al., 2022). The PEB chromophore was then manually docked into the MpeV/MpeB complex structure using Coot (Emsley and Cowtan, 2004).

## Results

### HPLC analyses of WH8020 PBS

**Figure 3:**
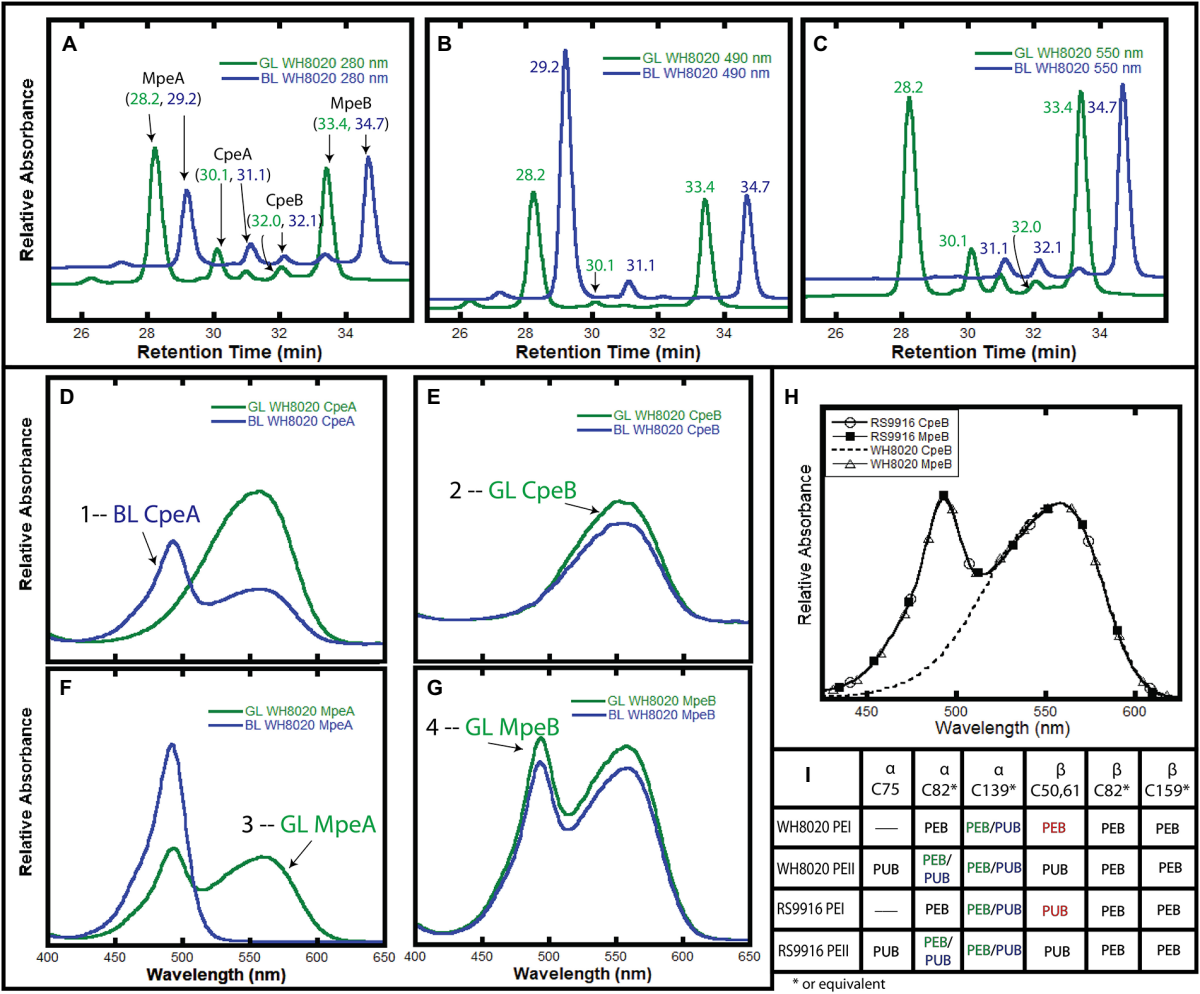
HPLC analysis of WH8020 PBS. **(A–C).** Chromatograms depicting relative absorbance of total protein (280 nm), presence of PUB (490 nm), and presence of PEB (560 nm). **(D–G)** Relative absorbance spectra of phycoerythrin I (PEI) and II (PEII) phycobiliprotein subunits separated by HPLC and denoted as follows: α-subunit of PEI (CpeA), β-subunit of PEI (CpeB), α-subunit of PEII (MpeA), β-subunit of PEII (MpeB). PBS extracted from WH8020 cells grown in an abundance of green light (GL, green lines) or blue light (BL, blue lines). **(H)** Comparison of the absorbance spectra of RS9916 and WH8020 β-subunits CpeB and MpeB. Note that WH8020 CpeB contains only PEB. **(I)** Table depicting the bilin content of these proteins at specific Cys residues (as inferred from Shukla et al., 2012 and Ong and Glazer, 1991). Sites that change for CA4 are shown as PEB (in green light) and PUB (in blue light) separated by a “/” The CpeB subunit differs at C50,61 between WH8020 and RS9916. Spectra numbered in **(D–G)** correspond with data in Table 1. These results are representative of two independent replicates.

Initially, we wanted to confirm that the bilin composition of PEI and PEII from wild type (WT) WH8020 PBS was as previously described (Ong and Glazer, 1991). Cells used in this previous study were isolated from cultures grown in white light, a light condition known to elicit a pigment phenotype equivalent to GL in CA4 strains (Ong and Glazer, 1991; Everroad and Wood, 2006; Humily et al., 2013). Here we isolated PBS from native WH8020 cells grown in two separate light conditions: GL for maximum PEB production and BL for maximum PUB production (Supplementary Figure 2). PBPs were then separated by HPLC with relative absorbance and chromatograms obtained using 550 nm (PEB) and 495 nm (PUB) for bilin content and 280 nm for total protein (Figures 3A–C; Shukla et al., 2012; Carrigee et al., 2020b). The numbered peaks in Figures 3D–G were collected, digested with trypsin, and analyzed by LC/MS/MS to identify the proteins present in each peak. As shown in Table 1, peaks labeled as MpeA, CpeA, CpeB, and MpeB were verified by MS coverage (Table 1; Figure 3). As expected for this CA4-A strain, variable bilin content was detected among the α-subunits depending on light quality (GL or BL), and the changes in PUB:PEB ratio observed in the spectra for CpeA and MpeA matched those documented for RS9916 during the CA4 process (Figures 3H,I; Kehoe, 2010; Shukla et al., 2012; Nguyen, 2018; Carrigee et al., 2020b). Unfortunately, we were not able to identify all bilins attached at each light condition for each protein by LC tandem MS, but we can infer the likely bilin content from our spectra and using the previous characterizations of WH8020 in white light (equivalent to GL; Ong and Glazer, 1991) and RS9916 in BL and GL (Shukla et al., 2012) as shown in Figure 3I.

**Table 1:** Mass spectrometry coverage of phycobilisome peptide fragments from WH8020. Trypsin digested samples of PEI α- (CpeA) and β-(CpeB) subunits and PEII α-(MpeA) and β-(MpeB) subunits showing their MS percent coverage, relative abundance (Rel Abund) and number (Num) of unique peptides found for each protein within each sample. Bold numbers for each sample correspond to numbered absorbance spectra in Figure 3.

1—CpeA sample			2—CpeB sample			3—MpeA sample			4—MpeB sample			Protein name
Num unique	% Cov	Rel Abund	Num Unique	% Cov	Rel Abund	Num Unique	% Cov	Rel Abund	Num Unique	% Cov	Rel Abund	
24	80.5	85.9%	17	66.5	4.4%	15	72	0.2%	8	55.5	0.1%	WH8020_CpeA
8	38.6	6.7%	9	46.7	54.9%	7	42.4	0.3%	10	36.4	2.7%	WH8020_CpeB
8	36.4	7.1%	6	36.4	3.7%	38	98.8	98.7%	11	78.2	0.5%	WH8020_MpeA
14	66.9	0.3%	11	51.7	37.0%	10	51.7	0.8%	28	96.6	96.7%	WH8020_MpeB

As expected from previous work showing that the CA4 process affects only MpeA and CpeA (Shukla et al., 2012), no changes were observed in the MpeB and CpeB proteins in GL vs. BL (Figures 3E,G). As previously reported by Ong and Glazer for WH8020 grown in white light (Ong and Glazer, 1991) and for RS9916 in both GL and BL (Shukla et al., 2012), WH8020 MpeB binds a doubly-linked PUB at C50, 61 and two PEB (C159 and C82) in a 1:2 ratio, or a PUB:PEB of ~0.58 independent of light condition (Figures 3E,G; Ong and Glazer, 1991). However, while RS9916 CpeB maintains bilin composition of one PUB (doubly-linked to C50, 61) and 2 PEB (C82 and C165) for CpeB regardless of growth conditions, HPLC analysis showed that WH8020 CpeB has PEB bound to all sites, including the doubly-linked C50, 61 position, consistent with what Ong and Glazer reported in 1991 (Ong and Glazer, 1991). The fraction collected for CpeB (Figure 3E, peak 2) only contains PEB (no PUB is detected) and no bilin content changes were detected in GL or BL.

### Comparative genomics analysis

MpeV was first suggested as a putative lyase by Wilbanks and Glazer, after sequencing of a large fraction of the PBS rod genomic region from WH8020 (Supplementary Figure 1; Wilbanks and Glazer, 1993). ClustalW analysis of protein sequences from RS9916 and WH8020 revealed 97.3% similarity between the CpeB substrates, 97.8% similarity between MpeB substrates, and 72.2% similarity between the MpeV homologs (Supplementary Figure 3). This led us to hypothesize that MpeV could be a lyase acting at C50, 61 on CpeB and perhaps also the lyase-isomerase acting on C50, 61 on MpeB in WH8020.

A comparison of *Synechococcus* CpeB sequences sorted by pigment type (PT) is shown in Supplementary Figure 4A [for review on PTs, see (Six et al., 2007; Humily et al., 2013; Grébert et al., 2022)]. All strains of *Synechococcus* belonging to PT2 (green light specialists with PEI and PEB only), PT3a (green light specialists with PEI, PEII, and a constitutively low PUB:PEB), about half of PT3dA (CA4-A) including WH8020, and the only representative of PT3eA (RCC307 that is genetically undistinguishable from typical PT3dA but exhibits only faint variations in the PUB:PEB ratio), all possess a H at position 141 in CpeB. In contrast, all strains that are either PT3c (typical BL specialists), PT3f (a rarer type of BL specialists), PT3dB (CA4-B), the other half of PT3dA including RS9916, and a natural mutant strain (BIOS-E4-1 that also display a BL specialist phenotype; Humily et al., 2013; Grébert et al., 2018) possess a L at position 141 in CpeB (Supplementary Figure 4A, blue highlights). By comparison for MpeB, all PT3a (or 3eA) strains exhibited a H whereas most other strains have a L, consistent with the fact that the former have a PEB and all others a PUB at C50, 61 (Supplementary Figure 4B). The only exception to this rule is PT3f strains which have a M at this position.

### Recombinant protein analysis

**Figure 4:**
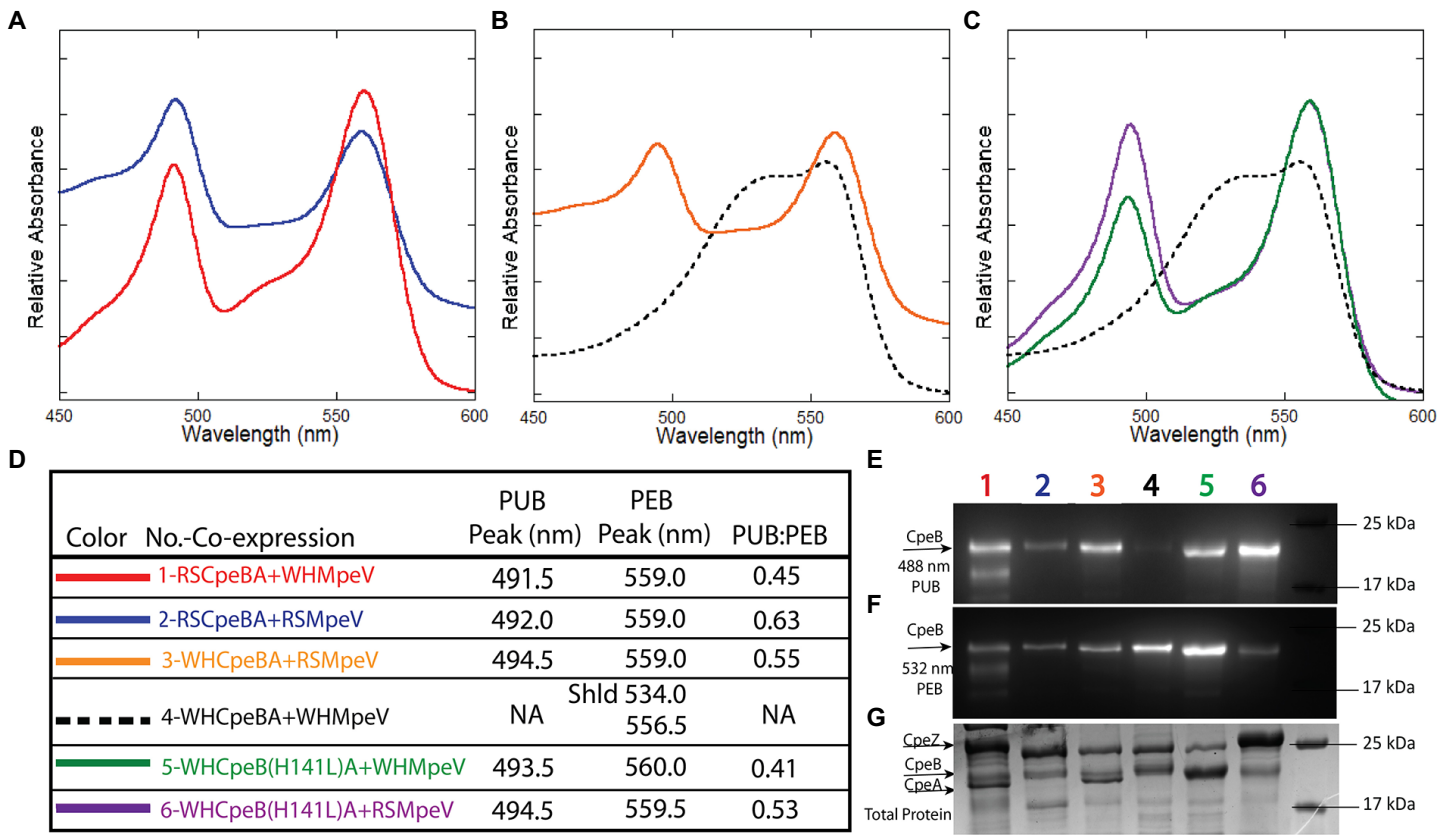
Absorbance spectra and SDS-PAGE for *Synechococcus* RS9916 and WH8020 CpeB co-expressions with MpeV. **(A–C)** Relative absorbance spectra depicting bilin addition to RSCpeB and WHCpeB when expressed in the presence of either WHMpeV or RSMpeV (as indicated in the legend in panel **D**). Purified protein substrates were resolved *via* SDS-PAGE and imaged with zinc-enhanced fluorescence at 460–490 nm **(E)** which excites PUB and at 520–545 nm **(F)** which excites PEB. Lanes are labeled with co-expression numbers and colors as listed in panel **D**. The same gel was then stained with Coomassie blue **(G)** to visualize proteins. All expressions contained RS9916 *cpeS*, *cpeZ*, and *ho1/pebS*. This study is representative of two independent replicates. NA means not applicable.

For simplicity, all recombinant proteins from RS9916 are hereafter prefixed with an “RS” and all WH8020 proteins are hereafter prefixed with a “WH” while generic referral to protein(s) from both strains will remain unprefixed (e.g., CpeA vs. RSCpeA or WHCpeA). We sought to determine the activity of WHMpeV compared to that of RSMpeV using our heterologous *E. coli* expression system with various substrates. All protein co-expressions analyzing β-subunits as substrates were designed to also express α-subunits in an effort to increase solubility of β-subunits as previously shown (Anderson and Toole, 1998; Kronfel et al., 2019b; Carrigee et al., 2020b). Our earlier work with RS9916 showed that the enzymes RSCpeS and RSCpeZ were required to obtain enough chromophorylated (attaching PEB at C82 of CpeB and MpeB), soluble RSCpeB and RSMpeB substrate to allow RSMpeV to function (Carrigee et al., 2020b). Therefore, these genes from RS9916 were expressed concomitantly in trials testing RSMpeV and WHMpeV activity (Figure 4). Even though detectable amounts of α-subunits (CpeA) co-purified with their respective β-subunits (CpeB; Figures 4E–G), CpeA was expectedly not chromophorylated by the available lyases (see section LC–MS–MS analyses of recombinant co-expressions below). CpeB was present in all co-expressions as determined by total protein staining using Coomassie blue (Figure 4G). Control co-expressions to assess bilin addition and site specificity included CpeB/CpeA expressed without a lyase, or expressed in the presence of RSCpeZ, RSCpeS, and/or MpeV as outlined in Supplementary Table 3. Absorbance spectra from recombinant co-expressions of RSMpeV or WHMpeV enzymes with RSCpeB, WHCpeB, or WHMpeB demonstrate that WHMpeV acts as a PEB lyase-isomerase, doubly ligating PUB on RSCpeB (Figure 4A, co-expression 1, red line; absorbance peak 491.5 nm) and WHMpeB (Supplementary Figure 5; absorbance peak at 493 nm), but it does not isomerize PEB when attaching it to WHCpeB (Figures 4B,C, co-expression 4, black dashed line). The addition of a doubly-ligated PEB on WHCpeB subunit is detected as a prominent shoulder at 534.0 nm in addition to a peak at 556.5 nm from the C82 PEB ligated by RSCpeS (Figures 4B,C, co-expression 4, black dashed line). This is consistent with observations by Kronfel et al. for a paralogous E/F type lyase called CpeF which doubly ligates a PEB on CpeB at an equivalent position in the freshwater cyanobacterium *Fremyella diplosiphon* (Kronfel et al., 2019b). We conclude that WHMpeV is capable of isomerizing activity on both WHMpeB and RSCpeB but that the WHCpeB substrate prevents the isomerization activity of WHMpeV.

### Analysis of WHMpeV enzymatic activity using structural modeling and site-directed mutagenesis

**Figure 5:**
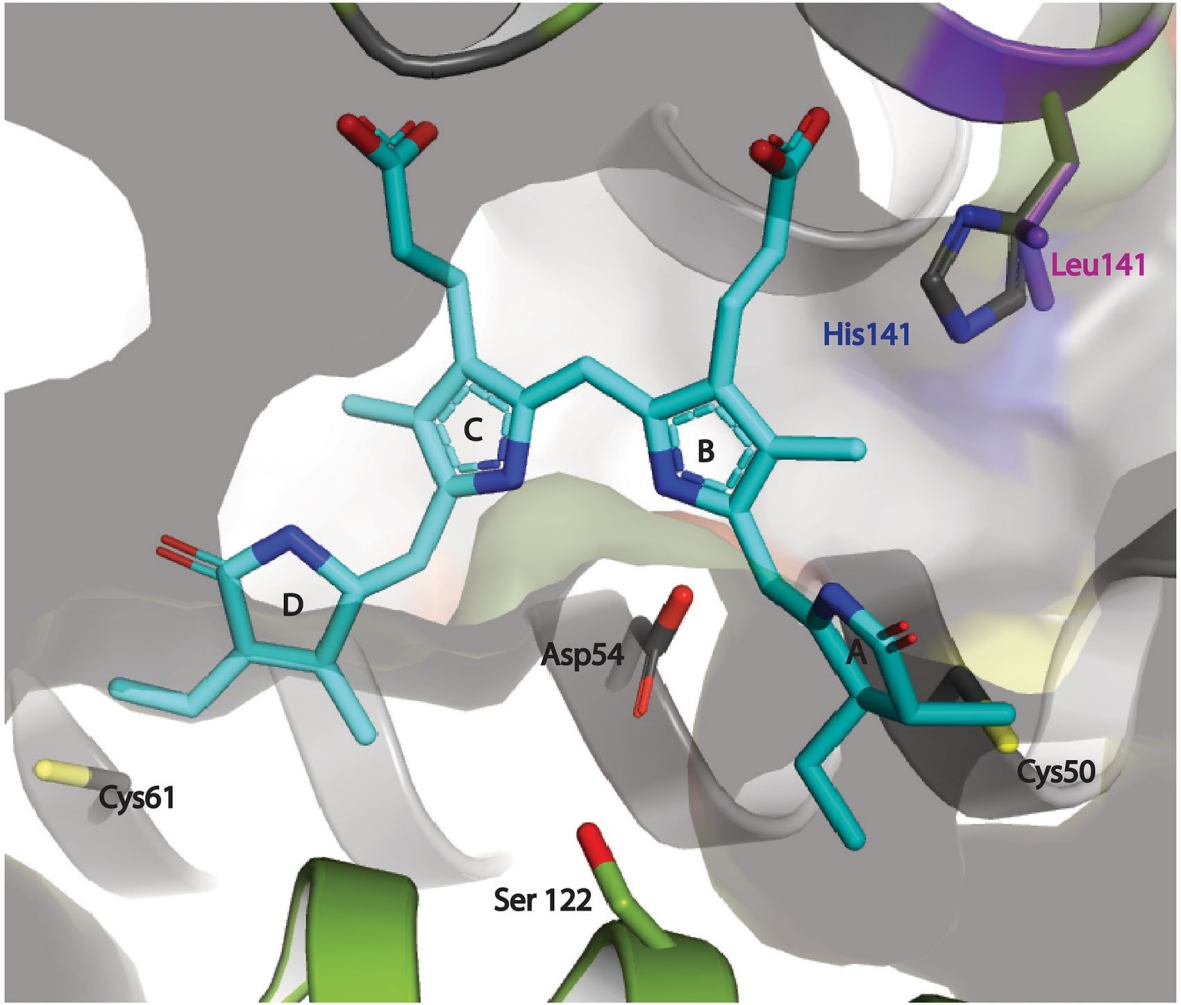
Docking of PEB at the putative binding cleft between the MpeV enzyme and WH8020 CpeB substrate. The PEB chromophore (cyan) is modeled in its final *anti, syn, anti-*conformation with ring A and ring D positioned next to their respective covalent anchors, (Cys50 and Cys61 of CpeB in yellow) bound between CpeB (gray ribbon), and MpeV (green ribbon). The pyrrole nitrogen atoms of rings B and C of PEB are stabilized *via* the side chain of the highly conserved Asp54 of CpeB. This model also places the side chain of His141 from WH8020 CpeB ~3.5 Å away from the ring B propionate group of PEB. We propose that this hydrogen bond between His141 and PEB potentially plays an important role in preventing the isomerization of PEB to PUB during the ligation reaction. The positioning of the conserved Ser122 of MpeV suggests it may be part of a putative active site for lyase activity.

As mentioned above, WHCpeB contains H at position 141 whereas RSCpeB contains L at this position (Supplementary Figures 3, 4). Molecular modeling of the structure of WHCpeB suggested that H141 should be positioned close to the C50, 61 bilin position and at ~7.4 Å from C50 (Figure 5). When PEB is docked with its ring A close to C50 and ring D close to C61, aspartic acid (D)54 of WHCpeB is poised toward the bilin, potentially interacting with the pyrrole nitrogen atoms of rings B and C (Figure 5). The arginine (R)57 residue on WHMpeV points toward the propionate chain of the C ring of PEB whereas MpeV-D116/R89 line the binding pocket near D ring (Figure 5B). Linkage at the A ring of PEB to C50 is a critical step in chromophore attachment, isomerization and stability for RSCpeB (Carrigee et al., 2020b). A site-directed mutation was introduced into WHCpeB converting H141 to leucine (H141L; Figure 5; Supplementary Table 1) to test whether this residue affected isomerization activity by WHMpeV. Absorbance data from the co-expressions including RSCpeZ, RSCpeS, and WHMpeV as outlined in Supplementary Table 3 revealed that this single substitution provided sufficient change(s) within the binding pocket to allow isomerization to occur (Figures 4, 5). Indeed, when native WHCpeB is expressed in the presence of RSCpeZ, RSCpeS, and WHMpeV, we see evidence of a doubly-ligated PEB at the C50, 61 position (Figures 4B–D, co-expression 4, black dashed line, and Table 2). However, when we perform this same co-expression using pHTCpeB(H141L)A (Supplementary Table 2), we see a doubly-linked PUB at the C50, 61 position (Figures 4C,D, co-expression 6 purple line, co-expression 5 green line, and Table 2). These findings indicate that the microenvironment of the WHCpeB binding pocket surrounding C50 plays a major role in WHMpeV isomerization activity at C50, 61.

**Table 2:** LC–MS–MS results for MpeV co-expressions. Extracted ion chromatograms were made using m/z 1200.8254 for 38–78 peptides with a bilin attached; 1053.7513 was extracted for the 38–78 peptide lacking a bilin. Sample abbreviations are defined in Supplementary Table 1.

Sample	38–78 peptide cysteine state^a^	Retention time^d^	EIC area^b^
WHCpeBA + RSCpeZ/CpeS, no MpeV	2B	N/D	N/D
	1B	N/D	N/D
	0B	22.34	3.34E+08
WHCpeBA + RSCpeZ/CpeS + WHMpeV	2B	20.94	2.35E+07
	1B	N/D	N/D
	0B	N/D	N/D
WHCpeBA + RSCpeZ/CpeS+ RSMpeV	2B	21.19	7.73E+08
	1B	N/D	N/D
	0B	22.6	1.08E+08
RSCpeBA + RSCpeZ/CpeS + WHMpeV^c^	2B	25.71,26.44	4.57E+07
	1B	27.25	3.59E+07
	0B	27.23	2.32E+07
WHCpeB(H141L)A + RSCpeZ/CpeS + RSMpeV^c^	2B	21.14	4.42E+09
	1B	23.2	6.79E+07
	0B	22.54	8.07E+07
WHCpeB(H141L)A + RSCpeZ/CpeS + WHMpeV^c^	2B	25.83,26.48	2.32E+08
	1B	27.27	2.41E+07
	0B	21.97	7.82E+07

Representative tandem mass spectra for each form of the peptide can be found in Supplementary Figures 5–7.

a The 38–78 peptide has C50, C61 and C73. Peptides with doubly-linked bilin bridging C50 and C61 are labeled 2B. Peptides with a singly-linked bilin at C50 are labeled 1B; peptides with no bilin but a disulfide between C60 and C73 are labeled 0B.

b All EICs were made using an 8 ppm mass window and a 3 point boxcar smoothing routine. Areas were integrated with the ICIS algorithm of Thermo QualBrowser 4.3.73.11. Results are representative of two independent replicates.

c Sample was run separately from the others using a slightly modified gradient.

d N/D means not detected.

### LC–MS–MS analyses of recombinant co-expressions

Table 2 shows peak areas obtained for extracted ion chromatograms (M + 4H)^4+^ ions of the tryptic peptide for CpeB containing residues 38–78. The peptide sequence is the same in both the RSCpeB and WHCpeB and is shown in Supplementary Tables 4–7. The negative control sample (WHCpeBA, no MpeV) shows no bilin attachment and interestingly, MS–MS data suggest C61 and C73 are linked by a disulfide bridge. Complete ligation of bilin to C50 and C61 was observed for the positive control sample (WHCpeB + WHMpeV). RSMpeV appears to modify WHCpeB and WHCpeB(H141L) at a slower rate than WHMpeV modifies WHCpeB as we observed unmodified, disulfide linked 38–78 peptide in the mixture. Intriguingly, the co-expressions where WHMpeV acted on RSCpeB or WHCpeB(H141L) showed evidence of bilins singly attached to the 38–78 peptide as well as normal double attachment and some unmodified substrate with no bilins attached at all. It is likely that the disulfide bond formed during purification and processing, as the cytoplasm of *E. coli* is generally a reducing environment (Gąciarz et al., 2017). However, its formation may indicate a lack of sufficient activity by MpeV or insufficient folding by CpeB to achieve the doubly ligated chromophore in some of these combinations (Table 2). Representative mass spectra supporting these observations comprise Supplementary Figures 5–7; Supplementary Tables 4–7, contain lists of tandem mass spectral fragment matches. In all samples containing RSCpeS, a bilin attached at C82 was observed, as previously described (Carrigee et al., 2020b). We did not observe any bilin modifications on CpeA in these co-expressions.

### Modeling of MpeV with substrates suggests the role of His141

To explore the possible role of CpeB H141 in conferring the lyase activity of MpeV, we built a structure model of MpeV in complex with two substrates: CpeB and PEB, using manual docking aided by AlphaFold2 implemented in the ColabFold server (Jumper et al., 2021; Mirdita et al., 2022; Figure 5). In this model, the bilin pigment adopts an *anti, syn, anti*-configuration in which the ring A and ring D are in close proximity of their respective C anchors (i.e., C50 and C61 of CpeB) and the pyrrole nitrogen atoms of PEB rings B/C are in hydrogen-bonding distances with CpeB-D54 (Figure 5). Interestingly, we note that the side chain at the 141 position of CpeB would directly interact with the ring B propionate (Figure 5). For example, in WH8020 CpeB, H141 potentially forms a hydrogen bond with the ring B propionate while L141 of RS9916 CpeB cannot. This difference may explain the absence of the isomerase activity of MpeV on the CpeB substrate of WH8020 because the hydrogen bond between H141 and ring B propionate group may confer steric hinderance for the bilin transformation required by the PEB to PUB isomerization during a ligation reaction (Yang et al., 2007).

## Discussion

Among the three clans of lyases, only some members of the E/F clan have been reported to have the capability to isomerize bilins during attachment (Everroad et al., 2006; Shukla et al., 2012; Humily et al., 2013; Sanfilippo et al., 2016); moreover very few studies regarding the mechanism among substrate-bilin-enzyme interactions during the isomerization reaction have been explored (Zhao et al., 2017; Kumarapperuma et al., 2022). The ability of a member of the E/F-type lyase family (in the present case MpeV) to behave as both a lyase and an isomerase to two very similar substrates has never been reported before and suggests the CpeB (and likely MpeB) substrate environment plays a role in the isomerization process. *Synechococcus* sp. WH8020 is a CA4-A capable strain whose β-subunits share a high sequence similarity with RS9916 β-subunits (Supplementary Figures 1, 3A); however, the doubly ligated bilin at C50, 61 of WHCpeB is a PEB (Ong and Glazer, 1991) rather than a PUB, as on RSCpeB (Shukla et al., 2012), matching the chromophore pattern that is seen *in vivo* in WH8020 PBS (Figure 4). We observed that PEB isomerization to PUB is possible on recombinant WHCpeB at the doubly ligated C50, 61 position as demonstrated when it was co-expressed with RSMpeV in *E. coli* (Figure 5B, co-expression 4, orange line; Table 2), leading us to conclude that the interaction(s) among the lyase, bilin, and β-PE substrate are all important for the isomerization process. These data suggest the WHCpeB substrate may play a role in blocking the isomerization by the WHMpeV bilin lyase during ligation. In examining the alignment between the RSCpeB and WHCpeB sequences and our molecular model of the WHCpeB C50, 61 binding pocket, we identified H141 as potentially important for affecting isomerization by preventing WHMpeV (but not RSMpeV) from isomerizing PEB to PUB under native conditions (Figures 3, 5). By altering WHCpeB with the H141L point mutation, we showed that WHMpeV was able to isomerize PEB to PUB at C50, 61 on this WHCpeB (H141L) mutant during the attachment reaction. One explanation for this phenomenon is steric hinderance of H141 within the binding pocket of WHCpeB due to its bulky side chain and its proximity to C50 on WHCpeB, the site of PEB A-ring attachment and subsequent location of the bond isomerization (Figure 5). Alternatively, H141 may be involved in protonation/deprotonation or hydrogen-bonding dependent activity affecting the bilin conformation within the binding pocket (Kumarapperuma et al., 2022). Our modeling suggested that H141 in CpeB is close to the ring B propionate of PEB. This type of interaction has been seen in bacteriophytochromes previously (Yang et al., 2007). H141 may hydrogen bond to this group, blocking the movement that has to occur during the isomerization reaction. L (or M) at this position would not be so constrained, allowing the isomerization to occur.

Historically, E/F-type lyases including MpeV exhibit broad variation in chromophore and PBP substrate specificity, while demonstrating high binding-site specificity (e.g., which C residue). Kumarapperuma and collaborators recently determined the crystal structure of the lyase-isomerase MpeQ, proposed a mechanism for the reaction, and compared it to the mechanism for the related PEB lyase MpeW (Kumarapperuma et al., 2022). For MpeQ, Tyrosine 318 was proposed to activate PEB, polarizing the C3 = C3^1^ double bond. The side chain of V319 creates a steric conflict with the A-ring of PEB. This conflict is resolved by the PEB/PUB isomerization reaction and then ligation can happen, resulting in the MpeA-PUB product. In the PEB lyase MpeW, ligation happens directly after Tyrosine 318 activates that bond because there is no steric hindrance by the smaller residue of G319 on MpeW. In the case of WHMpeV, we hypothesize that the H141 residue in WHCpeB hydrogen bonds to the ring B propionate, constraining the movement that may occur during the isomerization reaction, favoring ligation before isomerization can take place (Figures 2, 5). RSMpeV is capable of isomerizing WHCpeB (Figure 4B, orange line), so there must be subtle differences within the MpeV enzymes that also control this isomerization reaction. In order to better understand this, we would need a structure of the substrate CpeB with the enzyme MpeV and the substrate PEB, a goal we are currently working toward.

This H at 141 is conserved in all green light specialists, i.e., *Synechococcus* strains that have either no PUB (PT2) or a constitutively low PUB/PEB ratio (PT3a; Supplementary Figure 3; Six et al., 2007; Grébert et al., 2022). Instead of MpeV, these strains possess the PEB lyase CpeF, the most ancient member of the CpeF-MpeV-MpeU E/F lyase family, which ligates doubly-linked PEB at C50/61 (Kronfel et al., 2019b; Carrigee et al., 2020b). In contrast, the blue light specialists, i.e., PT3c and PT3f which constitutively have a high PUB/PEB ratio, and the PT3 dB strains, i.e., chromatic acclimators possessing a CA4-B island (Humily et al., 2013; Grébert et al., 2021), all have a L at CpeB-141, and we posit that these strains must have a PUB at C50, 61 of CpeB. All of them lack CpeF and MpeV, so we hypothesize that the lyase-isomerase necessary to bind a PUB at both CpeB and MpeB C50, 61 in these strains is MpeU, a partially characterized member of this E/F lyase family (Mahmoud et al., 2017; Carrigee et al., 2020b; Grébert et al., 2022). Interestingly, while all PT3dA strains, i.e., chromatic acclimators possessing a CA4-A island, possess MpeV, only half of them, including RS9916, MITS9220, BL107, PROS-9-1 and the natural mutant strain BIOS-E4-1 which has become a BL specialist by selective loss of *fciA*, *fciB*, and *mpeY* genes (Humily et al., 2013; Grébert et al., 2018), have L at CpeB-141, so we hypothesize that they all have PUB at C50, 61 of CpeB, as previously demonstrated for RS9916 (Shukla et al., 2012). The other half of the PT3dA strains, including WH8020, CC9311, BIOS-U3-1 and RCC307, which shows only faint variations of its PUB/PEB ratio (Humily et al., 2013) all have H at this 141 position and consequently must have PEB at CpeB 50–61, like was first shown in WH8020 by Ong and Glazer (1991) and checked by us in the present study. The identity of the amino-acid at position 141 is seemingly also important for MpeB, with all GL specialists that have a PEB at C50, 61 having a H, and all BL specialists and chromatic acclimators that have a PUB at C50, 61 having a L. The only exception to this rule is PT3f strains which have a M at the MpeB 141 position. Even though the phycobiliprotein chromophorylation of strains representative of this pigment type has never been formally determined, it is most likely that they bind a PUB at C50, 61 on both CpeB and MpeB. So, altogether it appears that the chromophore bound at C50, 61 of CpeB and/or MpeB in any PE-containing marine *Synechococcus* can be reliably predicted from the amino acid present at position 141 of these PE β-subunits.

Combined with the structural and mechanistic analyses previously performed on MpeQ (Kumarapperuma et al., 2022), the present study demonstrated that there are residues within the substrate that influence the isomerization reaction of lyase/isomerases, a novel finding. A complete understanding of the mechanisms of these isomerase-capable lyases within the E/F clan is a main challenge for future studies.

## Data availability statement

The original contributions presented in the study are publicly available. This data can be found at: MassIVE (https://massive.ucsd.edu/ProteoSAFe/static/massive.jsp), dataset ID number MSV000090019.

## Author contributions

LC and WS conceived of the study through discussions with the coauthors. WS supervised the work and together with LC wrote the draft manuscript. LC, JF, and XL conducted the recombinant protein analyses. LD and FP performed the growth and analyses of *Synechococcus* sp. WH8020 cells for PBS purification and performed the bioinformatic analyses of CpeB and MpeB. JK and JT performed the tandem mass spectrometry and analyzed the data. IT and XY modeled the structure of MpeV with CpeB and PEB. All authors contributed to the article and approved the submitted version.

## Funding

The Orbitrap Fusion Lumos was purchased with funds from the Precision Health Initiative of the Indiana University Bicentennial Grand Challenges Program. This research project has been supported by awards from the National Science Foundation to WS (MCB 2017171) and XY (MCB 2017274) and from the Agence Nationale de la Recherche (ANR) program EFFICACY (ANR-19-CE02-0019) to FP.

## Conflict of interest

The authors declare that the research was conducted in the absence of any commercial or financial relationships that could be construed as a potential conflict of interest.

## Publisher’s note

All claims expressed in this article are solely those of the authors and do not necessarily represent those of their affiliated organizations, or those of the publisher, the editors and the reviewers. Any product that may be evaluated in this article, or claim that may be made by its manufacturer, is not guaranteed or endorsed by the publisher.

## Footnotes

^1^ https://www.roscoff-culture-collection.org/

## Abbreviations

BL: Blue light
C: Cysteine residue
CA4: Type IV chromatic acclimation
CpeA/CpeB: α−/β-subunit of phycoerythrin type
I; EICs: Extracted ion chromatograms
GL: Green light
HT: Hexahistidine-tagged
H: Histidine
L: Leucine
MpeA/MpeB: α-/β-subunit of phycoerythrin type II
MS: Mass spectrometry
MW: Molecular weight
PAGE: Polyacrylamide gel electrophoresis
PBP: Phycobiliprotein(s)
PBS: Phycobilisome(s)
PDB: Protein Data Bank
PEI: Phycoerythrin I
PEII: Phycoerythrin II
PEB: Phycoerythrobilin
PUB: Phycourobilin
RS: *Synechococcus* sp. RS9916
SDS: Sodium dodecyl sulfate
WH: *Synechococcus* sp. WH8020.
