# Supplementary material for: The phycoerythrobilin isomerization activity of MpeV in Synechococcus sp. WH8020 is prevented by the presence of a histidine at position 141 within its phycoerythrin-I β-subunit substrate
Source: Front Microbiol. 2022 Nov 15;13:1011189. doi: 10.3389/fmicb.2022.1011189 (PMC9705338; doi:10.3389/fmicb.2022.1011189)
Supplement: Supplementary file 1 [file Data_Sheet_1.PDF]

Supplementary Material

1 Supplementary Figures and Tables

Supplementary Table 1: Primer sequences for *Synechococcus* strain WH8020 site directed mutagenesis of CpeB
Supplementary Table 2: Plasmids used in this study
Supplementary Table 3: Recombinant protein co-expressions and abbreviations
Supplementary Table 4: Observed tandem mass spectra peaks matching predicted fragment ions for peptide 38-78 with a bilin bridging cysteines 50 and 61 in Supplementary Figure 6.
Supplementary Table 5: Observed tandem mass spectra peaks matching predicted fragment ions for peptide 38-78 with no bilin and a disulfide bond bridging cysteines 61 and 73 in Supplementary Figure 7.
Supplementary Table 6: Observed tandem mass spectra peaks matching predicted fragment ions for peptide 38-78 with a bilin at position 50 and free thiols on cysteines 61 and 73 in Supplementary Figure 8B.
Supplementary Table 7: Observed tandem mass spectra peaks matching predicted fragment ions for peptide 38-78 with a bilin at position 50 and a disulfide bond bridging cysteines 61 and 73 in Supplementary Figure 8D.

Supplementary Figure 1: Map of the central part of the phycobilisome rod genomic region of *Synechococcus* sp. RS9916
Supplementary Figure 2: PUB:PEB excitation of WH8020 whole cells/PBS
Supplementary Figure 3: Predicted structure and amino acid sequence comparison of CpeB and MpeV proteins from *Synechococcus* strains WH8020 and RS9916
Supplementary Figure 4: Alignment of CpeB and MpeB sequences retrieved from 46 marine, phycoerythrin-containing *Synechococcus* strains
Supplementary Figure 5: *Synechococcus* strain WH8020 MpeV activity on WH8020 MpeB
Supplementary Figure 6: Representative extracted ion chromatogram, precursor mass spectrum, and tandem mass spectra for peptide 38-78 with a bilin bridging positions 50 and 61 from WHCpeB(H141L)A+ RSCpeZ/CpeS+ WHMpeV co-expression.
Supplementary Figure 7: Representative extracted ion chromatogram, precursor mass spectrum, and tandem mass spectra for peptide 38-78 with no bilin and a disulfide bridging positions 61 and 73 from WHCpeB(H141L)A+ RSCpeZ/CpeS+ WHMpeV co-expression.
Supplementary Figure 8: Representative extracted ion chromatogram, precursor mass spectra, and tandem mass spectra for three versions of peptide 38-78 with a singly attached bilin at position 50 from WHCpeB(H141L)A+ RSCpeZ/CpeS+ RSMpeV co-expression.

Supplementary Table 1 Primer sequences for *Synechococcus* strain WH8020 site directed mutagenesis of *cpeB*[+]. Overlapping extension PCR was used in conjunction with the listed primers to generate WH8020 HTCpeB(H141)A operon as previously described (Hussain and Chong, 2016). [+]Site-directed mutation in orange [*]GSP denotes gene specific primer and MP denotes mutant primer. [a] CpeBA operon used for generation of mutants was cloned into MCSI of *p*ET-DUET1 vectors containing resistance to ampicillin (AP). [b] Genes encoding hexahistidine-tags fused to protein product are indicated as HT

**Primer Name[*]**	**Sequence (5' to 3')[+]**	**Plasmid Name[a b]**
CpeBA.GSP.F.BamHI	CATAGGATCCGATGCTCGACGCATTCTCAC	pHTCpeBA
CpeBA.GSP.R.HindIII	CCGCCAGCTTTCAAGAGAGAGCATTGATAACATAAT	
CpeBA.MPH141L.F.CAC to CTC	GGCTTCCGCCACTGCTCTCATCGGAGAAAC	pHTCpeB(H141L)A
CpeBA.MPH141L.R.CAC to CTC	GTTGGTTTCTCCGATGAGAGCAGTGGCGG	
CpeBA.MPH141L.F.CAC to CTA	GGCTTCCGCCACTGCTCTAATCGGAGAAAC	
CpeBA.MPH141L.R.CAC to CTA	GTTGGTTTCTCCGATTAGAGCAGTGGCGG	
CpeBA.MPH141L.F.CAC to CTG	GGCTTCCGCCACTGCTCTGATCGGAGAAAC	
CpeBA.MPH141L.R.CAC to CTG	GTTGGTTTCTCCGATCAGAGCAGTGGCGG	
8020MpeB F (BamHI)	CAGGATCCCATGCTCGACGCATTCTCCAGGAAGGC	pHTMpeBA
8020MpeB R (EcoRI)	ATGAATTCAGATTCAGCTGATTGCGCTGATCACTG	
8020MpeA F (SalI)	AAAGTCGACAAGGAGACAACTCATGAAGTCTGTTATCACC	
8020MpeA R (HindIII)	AAAAAGCTTTCAACCAGGGAGTTGATCA	

Supplementary Table 2: Plasmids used in these studies

[a] Genes encoding hexahistidine-tags fused to protein product are indicated as "HT-"
[b] "NT-" is an abbreviation for "Non-Tagged" referring specifically to hexahistidine-tags
[c] Antibiotic resistance used to select for the presence of the plasmid (Ap: ampicillin; Cm: chloramphenicol; Km: kanamycin; Sp: spectinomycin)

**Plasmid name[a]**	Recombinant proteins produced[b]	Parent vector	Antibiotic[c]	Reference
**pHTCpeA/HTCpeB**	*Synechococcus* sp. RS9916 HT-CpeA/HT-CpeB	pETDuet-1	Ap	(Carrigee et al., 2020a; Carrigee et al., 2020b)
**pHTCpeZ**	*Synechococcus* sp. RS9916 HT-CpeZ	pCOLADuet-1	Km	(Carrigee et al., 2020a)
**pHTMpeV**	*Synechococcus* sp. RS9916 HT-MpeV	pCDFDuet-1	Sp	(Carrigee et al., 2020b)
**pHTCpeZ/CpeS**	*Synechococcus* sp. RS9916 HT-CpeZ/NT CpeS	pCOLADuet-1	Km	(Carrigee et al., 2020b)
**pHTCpeA/HTMpeB**	*Synechococcus* sp. RS9916 HT-MpeA/HT-MpeB	pETDuet-1	Ap	(Carrigee et al., 2020b)
**pHTCpeBA**	*Synechococcus* sp. WH8020 HT-CpeB/NT CpeA	pETDuet-1	Ap	This study
**pHTCpeB(H141L)/A**	*Synechococcus* sp. WH8020 HT-CpeB (H141L)/NT CpeA	pETDuet-1	Ap	This study
**pHTMpeBA**	*Synechococcus* sp. WH8020 HT-MpeB/NT MpeA	pETDuet-1	Ap	This study
**pHTMpeV2**	*Synechococcus* sp. WH8020 HT-MpeV	pCDFDuet-1	Sp	This study
**pPebS**	Myovirus Ho1 and NT-PebS	pACYCDuet-1	Cm	(Dammeyer et al., 2008; Kronfel et al., 2019)

Supplementary Table 3 Recombinant protein co-expressions and abbreviations [a]HT indicates the presence of a hexa-histidine tag fused to protein product. [b]Lyase enzymes expressed from clones of RS9916 (RS) and WH8020 (WH) genes. [c]Expressed with PebS/HoI to generate PEB from *E. coli'*s heme. [d]Co-expressions performed with and without RSCpeS/RSCpeZ as previously described (Carrigee et al., 2020b)

**PE subunit(s)[a]**	**Lyase(s)[b]**	**Recombinant co-expression abbreviations[c]**
**WH8020**		
HTCpeB/CpeA	--	WHCpeBA
	(WH) HTMpeV	WHCpeBA+WHMpeV[d]
		WHCpeBA+WHMpeV+ RSCpeS/RSCpeZ
	(RS) HTMpeV	WHCpeBA+RSMpeV[d]
		WHCpeBA+RSMpeV+ RSCpeS/RSCpeZ
HTCpeB(H141L)/CpeA	--	WHCpeB(H141L)A
	(WH) HTMpeV	WHCpeB(H141L)A+WHMpeV[d]
		WHCpeB(H141L)A+WHMpeV+ RSCpeS/RSCpeZ
	(RS) HTMpeV	WHCpeB(H141L)A+RSMpeV[d]
		WHCpeB(H141L)A+RSMpeV+ RSCpeS/RSCpeZ
HTMpeB/MpeA	(WH) HTMpeV	WHMpeBA+WHMpeV+ RSCpeS/RSCpeZ
**RS9916**		
HTCpeA/HTCpeB	--	RSCpeBA
	(WH) HTMpeV	RSCpeBA+WHMpeV[d]
		RSCpeBA+WHMpeV+ RSCpeS/RSCpeZ
	(RS) HTMpeV	RSCpeBA+RSMpeV[d]
		RSCpeBA+RSMpeV+ RSCpeS/RSCpeZ

Supplementary Table 4: Observed tandem mass spectra peaks matching predicted fragment ions for peptide 38-78 with a bilin bridging cysteines 50 and 61 in Supplementary Figure 6C- D. Observed masses are the average of those seen in the tandem mass spectra of the +3 and +4 precursors; ions in brown were only observed in the +4 ion tandem MS. Italicized masses came from the precursor scan. Sequence of peptide from residues 38-78.

LDAVNAITSNASC$_{50}$IVSDAVTGMIC$_{61}$ENTGLIQAGGNC$_{73}$YPNRR

Blue letters indicate observed b- or a-type fragment ions; red letters correlate to observed y-type fragments, purple letters indicate sites where both b- and y-type ions were observed. These data suggest a bilin crosslinking Cysteine 50 to Cysteine 61.

m/z observed	theoretical m/z	Difference (PPM)	Putative ID	m/z observed	theoretical m/z	Difference (PPM)	Putative ID
201.1229	201.1234	-2.5	a2	731.3687	731.3723	-5.0	y13^{2+}
229.1180	229.1183	-1.5	b2	759.8812	759.8833	-2.8	y14^{2+}
300.1546	300.1554	-2.7	b3	798.4314	798.4356	-5.3	b8
343.1647	343.1652	-1.5	BC rings PUB	810.4037	810.4072	-4.3	y15^{2+}
371.2285	371.2289	-1.1	a4	867.4271	867.4286	-1.8	y16^{2+}
399.2235	399.2238	-0.8	b4	931.9477	931.9499	-2.4	y17^{2+}
450.2036	450.2023	2.9	ABC rings PUB	960.4611	960.4605	0.5	(M+5H)$^{5+}$
464.2177	464.218	-0.8	BCD rings PUB	1019.4506	1019.4476	2.9	y9-NH3
496.2399	496.2402	-0.7	b5-NH3	1036.4747	1036.4741	0.6	y9
513.2666	513.2667	-0.3	b5	1070.5397	1070.5477	-7.5	b11
554.2582	554.2593	-2.1	y10^{2+}	1200.3253	1200.3238	1.2	(M+4H)$^{4+}$
556.3068	556.3089	-3.8	a6	1214.5633	1214.5718	-7.0	y29^{3+}
567.2771	567.2777	-1.1	b6-NH3	1218.5410	1218.5433	-1.9	y11-NH3
584.3024	584.3039	-2.6	b6	1235.5757	1235.5698	4.7	y11
587.2853	587.2864	-1.9	ABCD rings PUB	1243.5723	1243.5825	-8.2	y30^{3+}
609.7765	609.7753	1.9	y11-NH3^{2+}	1267.2508	1267.2615	-8.4	y31^{3+}
618.2879	618.2885	-1.1	y11^{2+}	1305.2657	1305.2758	-7.7	y32^{3+}
652.3655	652.3665	-2.1	a7-NH3	1367.9575	1367.969	-8.4	y34^{3+}
674.8293	674.8306	-1.9	y12^{2+}	1405.6511	1405.6637	-9.0	y35^{3+}
680.3609	680.3614	-0.8	b7-NH3	1600.0967	1600.0959	0.5	(M+3H)$^{3+}$
697.3861	697.3879	-2.7	b7				

Supplementary Table 5: Observed tandem mass spectra peaks from Supplementary Figure 7C-D matching predicted fragment ions for peptide 38-78 with no bilin and a disulfide bridging cysteines 61 and 73. Observed masses are the average of those seen in the tandem mass spectra of the +3 and +4 precursors. Ions in green were only observed in the +4 ion tandem MS; ions in brown were only seen in the +3 tandem ion mass spectrum. Italicized ions were observed in the precursor mass spectrum. Sequence of peptide from residues 38-78.
LDAVNAITSNASC$_{50}$IVSDAVTGMIC$_{61}$ENTGLIQAGGNC$_{73}$YPNRR
Blue letters indicate observed b- or a-type fragment ions; red letters correlate to observed y-type fragments, purple letters indicate sites where both b- and y-type ions were observed. These data suggest a bilin crosslinking Cysteine 50 to Cysteine 61.

m/z observed	theoretical m/z	Difference (PPM)	Putative ID	notes	m/z observed	theoretical m/z	Difference (PPM)	Putative ID	notes
201.122	201.1234	-7.0	a2		1070.5416	1070.5477	-5.7	b11	
229.1169	229.1183	-6.1	b2		1104.5064	1104.5090	-2.4	$y20^{2+}$	S-S 61-73
300.1537	300.1554	-5.8	b3		1133.0123	1133.0197	-6.5	$y21^{2+}$	S-S 61-73
331.2193	331.2201	-2.4	y2		1157.5864	1157.5797	5.8	b12	
371.2267	371.2289	-5.9	a4		1183.5364	1183.5436	-6.1	$y22^{2+}$	S-S 61-73
399.2215	399.2238	-5.8	b4		1233.0682	1233.0778	-7.8	$y23^{2+}$	S-S 61-73
496.2387	496.2402	-3.1	b5-NH3		1260.0814	1260.0830	-1.3	$y24^{2+}$-NH3	S-S 61-73
513.2643	513.2667	-4.8	b5		1260.5814	1260.5889	-5.9	b13	no S-S bond
542.3112	542.3158	-8.6	y4		1268.5871	1268.5963	-7.3	$y24^{2+}$	S-S 61-73
567.2758	567.2769	-2.0	b6-NH3		1326.1023	1326.1098	-5.7	$y25^{2+}$	S-S 61-73
584.3006	584.3039	-5.6	b6		1369.6187	1369.6258	-5.2	$y26^{2+}$	S-S 61-79
680.3584	680.3614	-4.4	b7-NH3		1373.6642	1373.6729	-6.3	b14	no S-S
697.3839	697.3879	-5.7	b7		*1403.9976*	*1403.9977*	*-0.1*	*$(M+3H)^{3+}$*	
798.4291	798.4356	-8.2	b8		1419.1520	1419.1600	-5.7	$y27^{2+}$	S-S 61-79
982.4498	982.4467	3.1	$y18^{2+}$	S-S 61-73	1475.6955	1475.7020	-4.4	$y28^{2+}$	S-S 61-73
1038.9839	1038.9887	-4.6	$y19^{2+}$	S-S 61-73	1527.1990	1527.2066	-5.0	$y29^{2+}$	1 S-S bond
1053.5269	1053.5211	5.5	b11-NH3		1570.7132	1570.7226	-6.0	$y30^{2+}$	1 S-S bond
1053.2505	*1053.2501*	*0.4*	*$(M+4H)^{4+}$*		1597.7271	1597.7279	-0.5	$y31^{2+}$-NH3	1 S-S bond

Supplementary Table 6: Observed masses from tandem mass spectrum in Supplementary Figure 8-B matching fragments predicted for peptide 38-78 with a bilin attached to position 50 and free thiols on Cysteines 61 and 73. Italicized ions were observed in the precursor mass spectrum. Sequence of 38-78: LDAVNAITSNASC$_{50}$IVSDAVTGMIC$_{61}$ENTGLIQAGGNC$_{73}$YPNRR Blue letters indicate residues for which a- or b-type fragment were observed; red letters indicate residues for which y-ions were observed.

m/z observed	theoretical m/z	Difference (PPM)	Putative ID	m/z observed	theoretical m/z	Difference (PPM)	Putative ID
201.1228	201.1234	-3.0	a2	618.287	618.2885	-2.4	y11^{2+}
229.1178	229.1183	-2.2	b2	652.3654	652.3665	-1.7	a7-NH3
272.1604	272.1605	-0.4	a3	669.3877	669.3930	-7.9	a7
282.145	282.1448	0.7	b3-H2O	674.8303	674.8306	-0.4	y12^{2+}, no disulf
300.1552	300.1554	-0.7	b3	680.3611	680.3614	-0.4	b7-NH3
343.1643	343.1652	-2.6	BC rings bilin	697.3863	697.3879	-2.3	b7
371.2283	371.2289	-1.6	a4	731.3692	731.3726	-4.6	y13^{2+}, no disulf
381.2138	381.2132	1.6	b4-H2O	759.8796	759.8833	-4.9	y14^{2+}, no disulf
399.2233	399.2238	-1.3	b4	781.4106	781.4090	2.0	b8-NH3
464.2176	464.218	-0.9	BCD rings bilin	798.4341	798.4356	-1.9	b8
495.2558	495.2562	-0.8	b5-H2O	810.4011	810.4072	-7.5	y15^{2+}, no disulf
496.2405	496.2402	0.6	b5-NH3	867.4269	867.4286	-2.0	y16^{2+}, no disulf
513.2659	513.2667	-1.6	b5	931.9456	931.9499	-4.6	y17^{2+}, no disulf
539.2823	539.2824	-0.2	a6-NH3	*960.4610*	*960.4605*	*0.5*	*(M+5H)$^{5+}$, no disulf*
542.3143	542.3158	-2.8	y4	1019.4497	1019.4476	2.1	y9-NH3, no disulf
554.2594	554.2593	0.2	y10^{2+}	1036.4748	1036.4741	0.7	y9, no disulf
556.3072	556.3089	-3.1	a6	1039.9924	1039.9966	-4.0	y19^{2+}, no disulf
566.2925	566.2933	-1.4	b6-H2O	1105.5084	1105.5168	-7.6	y20^{2+}, no disulf
567.2776	567.2773	0.5	b6-NH3	1134.0211	1134.0275	-5.6	y21^{2+}, no disulf
584.3027	584.3039	-2.1	b6	1184.5473	1184.5514	-3.5	y22^{2+}, no disulf
587.2851	587.2864	-2.2	ABCD rings bilin	*1200.3242*	*1200.3238*	*0.3*	*(M+4H)$^{4+}$, no disulf*
609.7748	609.7753	-0.8	y11-NH3^{2+}, no disulf	*1600.0976*	*1600.0959*	*1.1*	*(M+3H)$^{3+}$, no disulf*

Supplementary Table 7: Observed tandem mass spectra peaks from Supplementary Figure 8D matching predicted fragment ions for peptide 38-78 with a bilin at position 50 and a possible disulfide bond bridging cysteines 61 and 73. Red lines show ions that indicate a disulfide present between positions cysteines 61 and 73. Italicized ions were observed in the precursor mass spectrum. Sequence of 38-78. LDAVNAITSNASC$_{50}$IVSDAVTGMIC$_{61}$ENTGLIQAGGNC$_{73}$YPNRR
Blue letters indicate residues for which a- or b-type fragment were observed; red letters indicate residues for which y-ions were observed. These data indicate this LC peak contained a mixture of disulfide and free-thiol versions of the peptide.

m/z observed	theoretical m/z	Difference (PPM)	Putative ID	m/z observed	theoretical m/z	Difference (PPM)	Putative ID
201.1225	201.1234	4.5	a2	680.3626	680.3614	-1.8	b7-NH3
229.1177	229.1183	2.6	b2	697.3872	697.3879	1.0	b7
272.1615	272.1605	-3.7	a3	780.4238	780.4250	1.5	b8-H2O
282.1444	282.1448	1.4	b3-H2O	781.4095	781.4090	-0.6	b8-NH3
300.1553	300.1554	0.3	b3	798.4315	798.4356	5.1	b8
343.1648	343.1652	1.2	BC rings bilin	931.9496	931.9499	0.3	y17^{2+} no disulf
371.2287	371.2289	0.5	a4	*960.0608*	*960.0573*	*-3.6*	*(M+5H)$^{5+}$, disulf*
399.2244	399.2238	-1.5	b4	*960.4592*	*960.4605*	*1.4*	*(M+5H)$^{5+}$, no disulf*
464.2179	464.2180	0.2	BCD ring bilin	1039.9992	1039.9966	-2.5	y18^{2+} no disulf
496.2413	496.2402	-2.2	b5-NH3	1070.5476	1070.5477	0.1	b11
513.2664	513.2667	0.6	b5	1105.5149	1105.5168	1.7	y20^{2+} no disulf
556.3087	556.3089	0.4	a6	1133.0189	1133.0197	0.7	y21^{2+}, disulf
567.2787	567.2779	-1.4	b6-NH3	1134.0240	1134.0275	3.1	y21^{2+}, no disulf
584.3043	584.3039	-0.7	b6	1183.5480	1183.5436	-3.7	y22^{2+}, disulf
587.2874	587.2864	-1.7	ABCD rings bilin	1184.5504	1184.5514	0.8	y22^{2+}, no disulf
618.2868	618.2885	2.7	y11^{2+} no disulf	*1199.8178*	*1199.8198*	*1.7*	*(M+4H)$^{4+}$, disulf*
669.3863	669.3930	10.0	a7	*1200.3217*	*1200.3238*	*1.7*	*(M+4H)$^{4+}$, no disulf*
679.3775	679.3774	-0.1	b7-H2O				

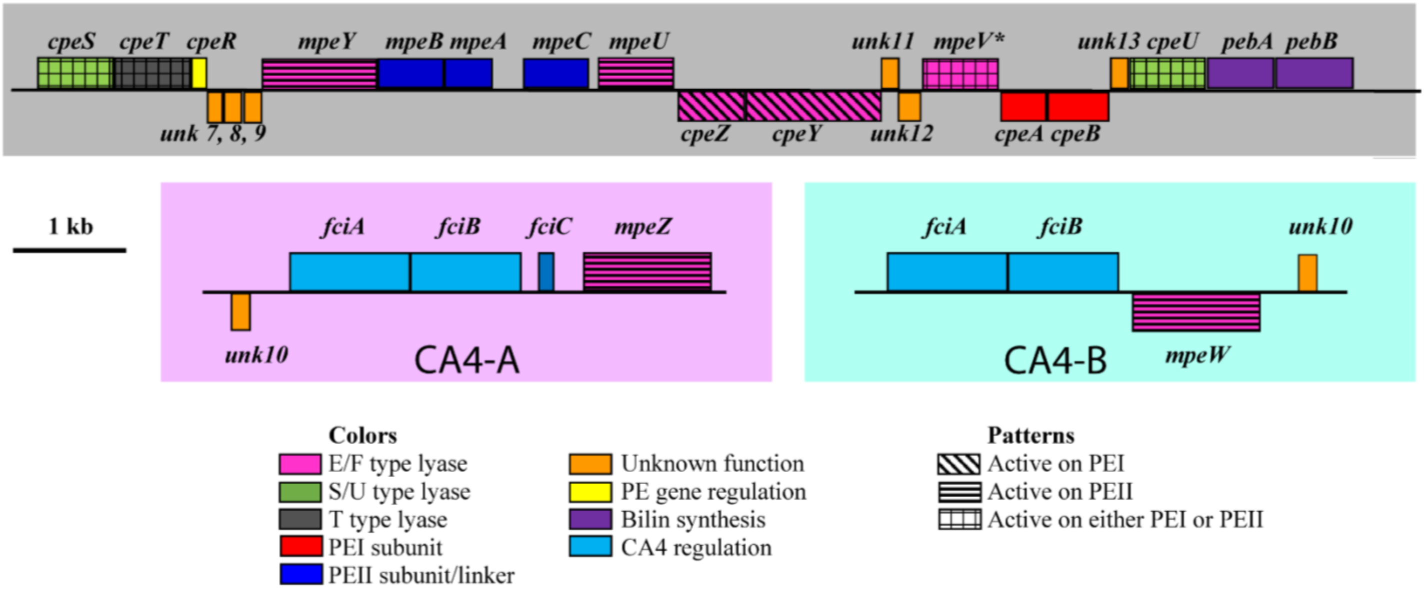

Supplementary Figure 1. Map of the central part of the phycobilisome rod genomic region of *Synechococcus* spp. The physical map highlighted in the grey box includes the operons encoding the PEI and PEII subunits, all phycoerythrin-related lyase genes, one PEII linker and a number of genes of yet unknown function (Six et al., 2007). Note that lyase genes present in the subregion extending from *unk7* to *mpeC* are specific to PEII, while those present in the rest of the region are either specific of PEI or can act on both PEI and PEII (Wilbanks and Glazer, 1993; Six et al., 2007; Shukla et al., 2012). A map depicting the CA4 genomic islands and regulatory genes in configuration A (CA4-A, fuchsia box) on the left and configuration B (CA4-B, light blue box) on the right (Humily et al., 2013; Sanfilippo et al., 2016; Sanfilippo et al., 2019). Adapted from (Six et al., 2007; Humily et al., 2013; Carrigee et al., 2020b).

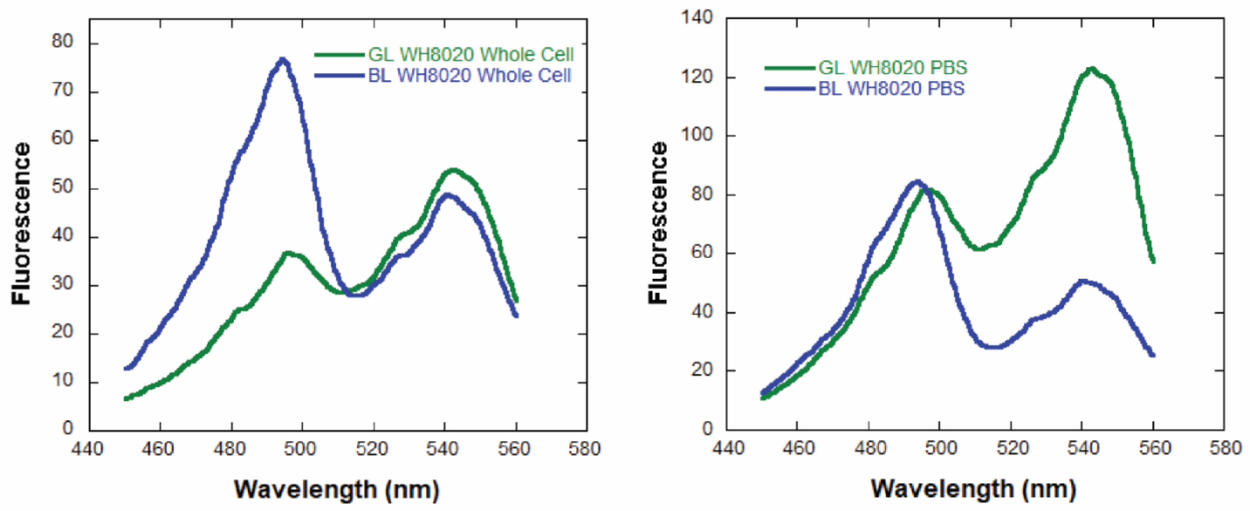

Supplementary Figure 2 PUB:PEB excitation of WH8020 whole cells/PBS. Relative fluorescence of whole cell *Synechococcus* sp. WH8020 wild type cells (left) and PBS purified via sucrose gradient (right). Spectra represent cells grown in blue light (BL, blue lines) and green light (GL, green lines) with PUB peak present at 495 nm and PEB peak at 545 nm.

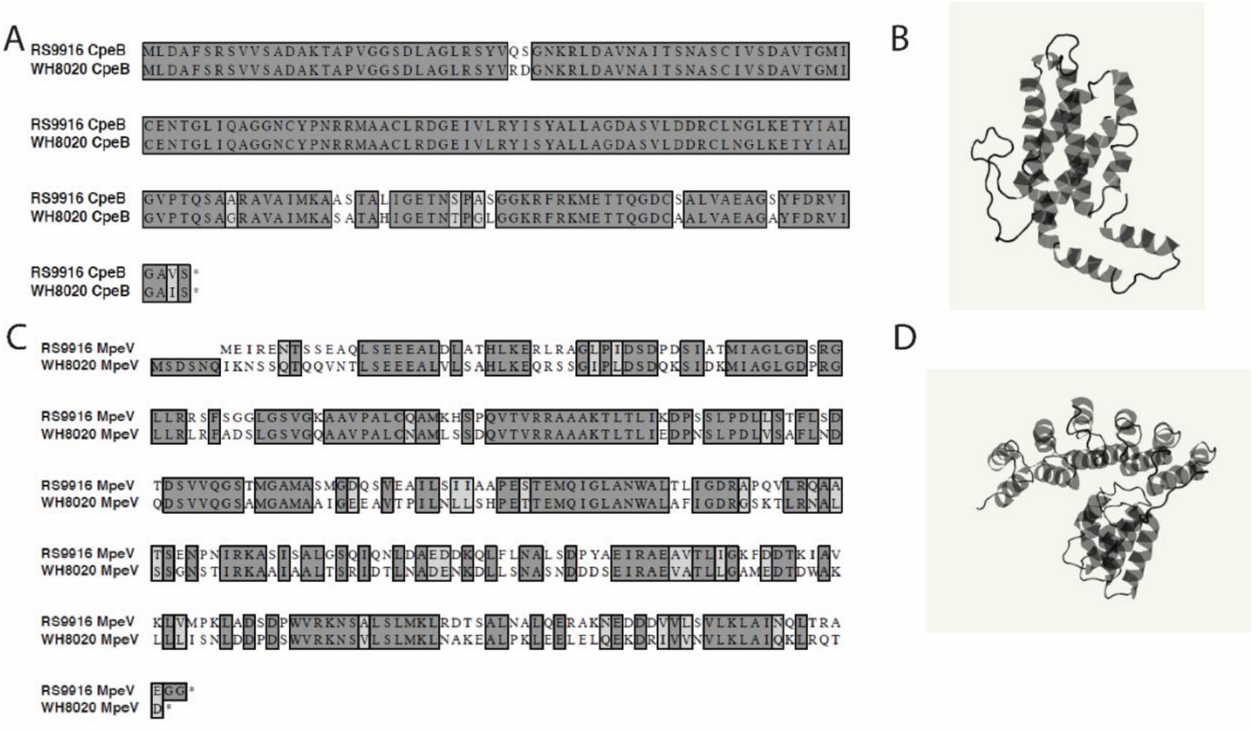

Supplementary Figure 3. Predicted structure and amino acid sequence comparison of CpeB and MpeV proteins from *Synechococcus* strains WH8020 and RS9916. **A** Clustal W comparison of the amino acid sequences of CpeB. **B** Depiction of Phyre^2 predicted structure of CpeB substrate proteins from RS9916 and WH8020 using the closest characterized structure PDB: d1b8db_ (Ritter et al., 1999). **C** Clustal W comparison of the amino acid sequences of MpeV. **D** Depiction of Phyre^2 predicted structure of the paralogous lyase/lyase isomerase MpeV between strains using the closest characterized structure PDB: 6fsq (Léger et al., 2019). Clustal W shading schemes as follows: identities are indicated with dark shading and similarities with light shading.

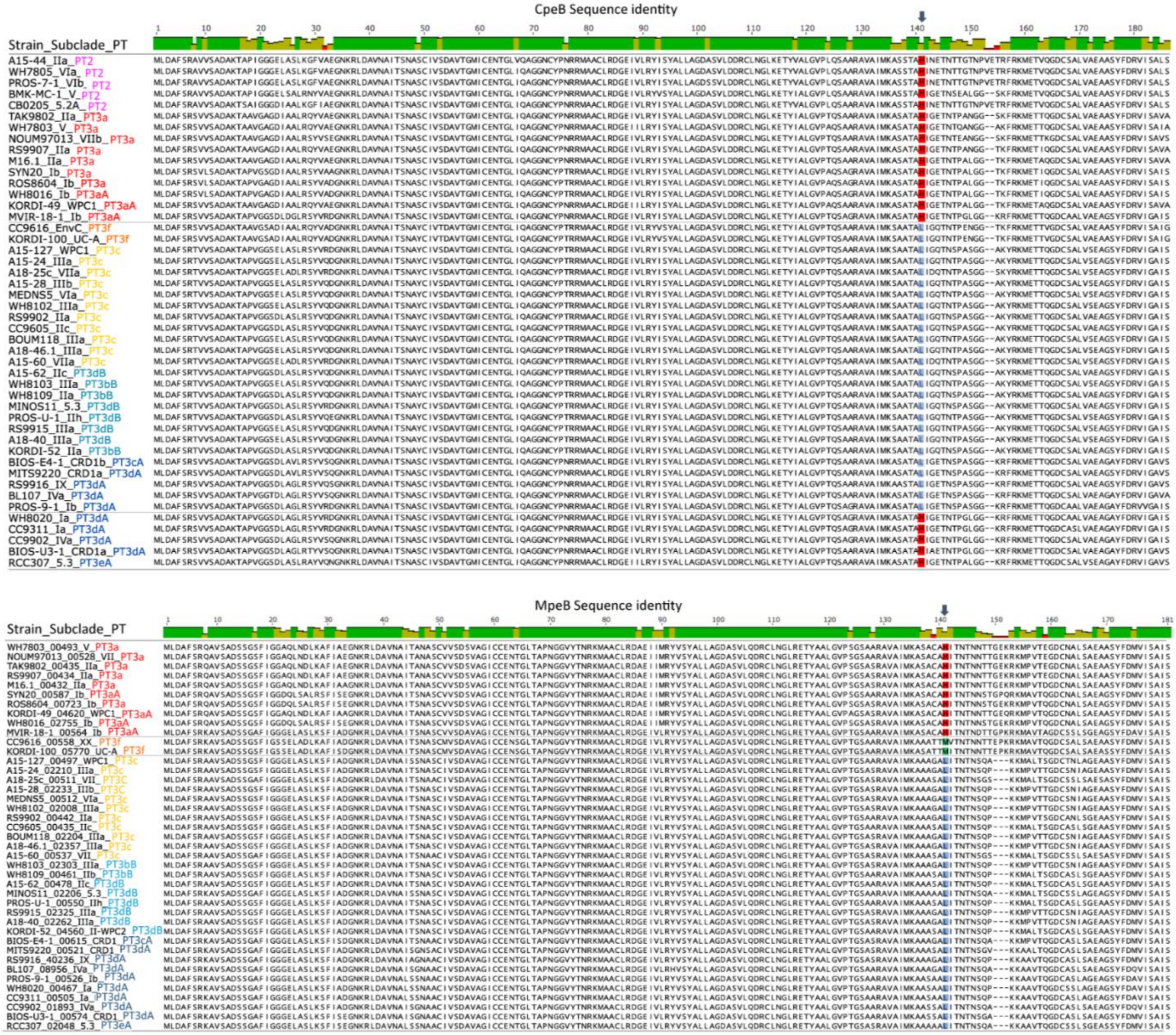

Supplementary Figure 4: Alignments of CpeB and MpeB sequences retrieved from marine *Synechococcus* spp. CpeB sequence alignments and MpeB sequence alignments are shown. Strains have been ordered by pigment type (PT) *sensu* (Humily et al., 2013), as indicated by a different color after the strain name and corresponding subclade (or subcluster for strains RCC307 and MINOS11). The top banner of each alignment indicates the sequence identity at each position (100% identical sites are shown in green). An arrow indicates position 141, at which strains have a histidine (red background), leucine (blue background) or methionine (green background, in MpeB from Pt3f strains only) residue. Sequences have been retrieved from the Cyanorak database [(Garczarek et al., 2021); http://sb-roscoff.fr/cyanorak].

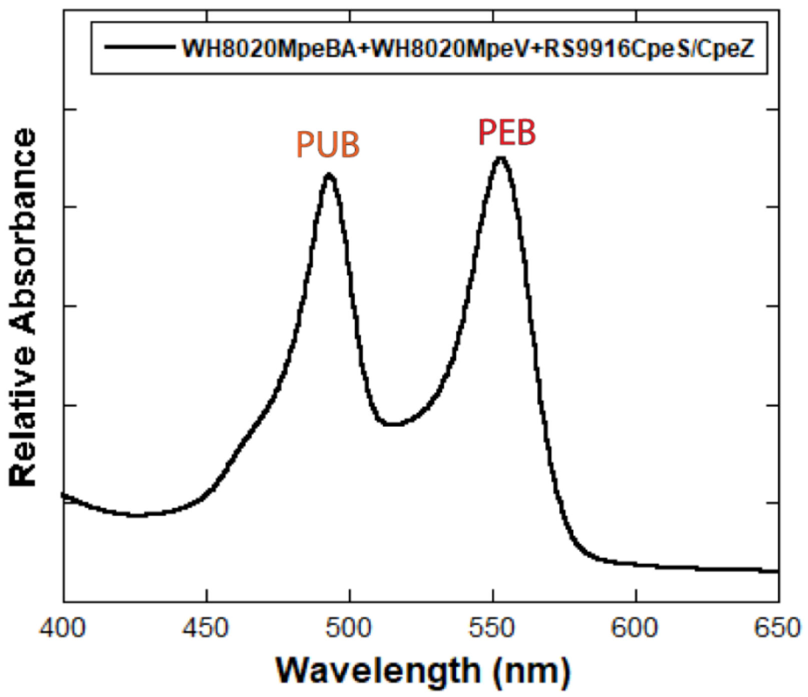

Supplementary Figure 5: *Synechococcus* strain WH8020 MpeV activity on WH8020 MpeB. Relative absorbance depicting enzymatic addition of bilin to WH8020 MpeB by WH8020 MpeV (PUB at 493 nm) and RS9916 CpeZ/CpeS (PEB at 553 nm). This study is representative of two independent replicates.

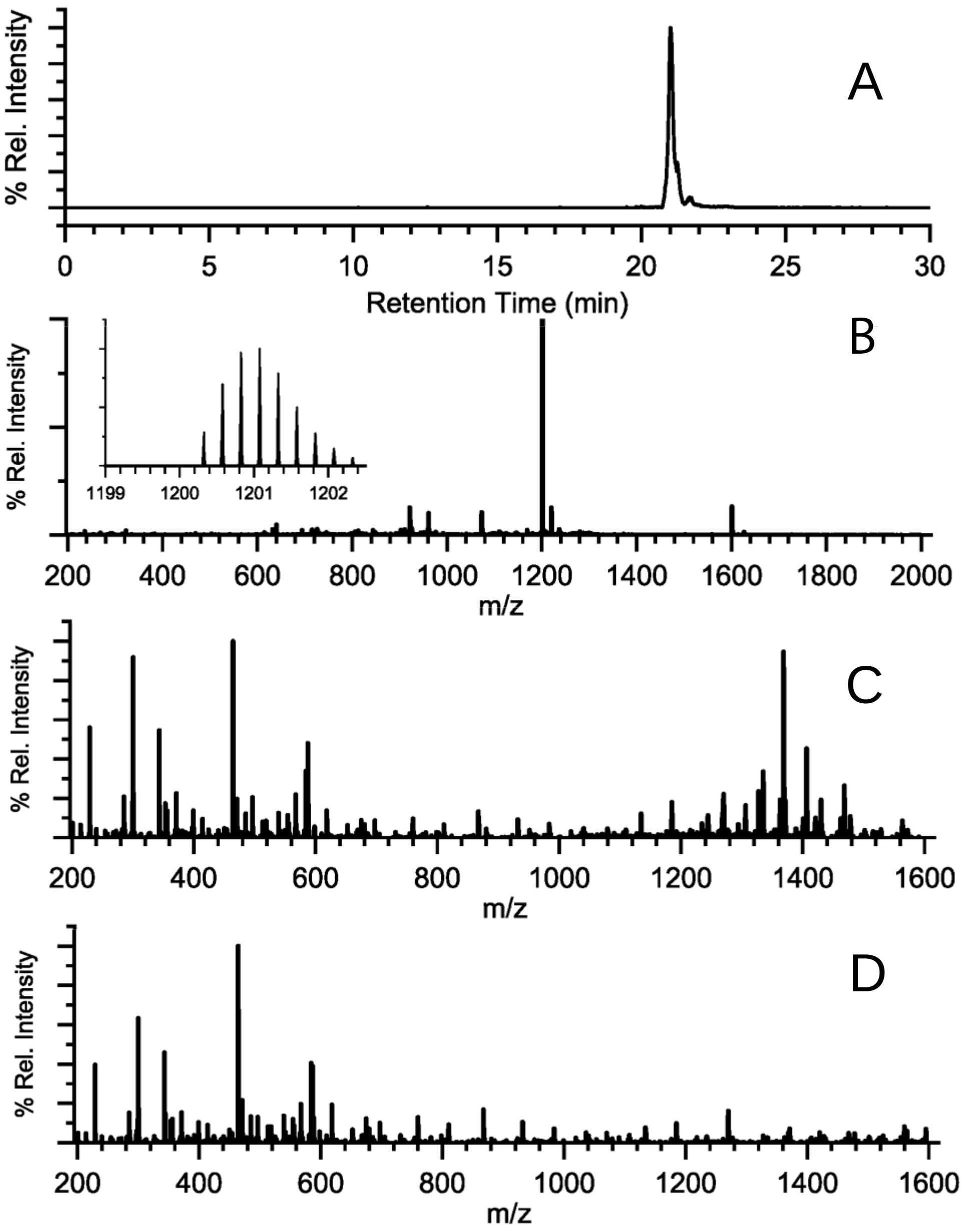

Supplementary Figure 6: Representative extracted ion chromatogram, precursor mass spectrum, and tandem mass spectra for peptide 38-78 with a bilin bridging positions 50 and 61 from WHCpeB(H141L)A+ RSCpeZ/CpeS+ RSMpeV co-expression. A. Extracted Ion Chromatogram for precursor ion at m/z 1200.8265 (most intense isotope in pattern); peak apex is at 21.06 minutes. B. Mass Spectrum of the 21.06 minute peak; inset shows $(M+4H)^{4+}$ isotopic cluster. C. Tandem mass spectrum of m/z 1200.82 @ 21.06 minutes. D. Tandem mass spectrum of m/z 1600.80 @ 21.06 minutes. Bottom: Sequence of peptide from residues 38-78. Blue letters indicate observed b- or a-type fragment ions; red letters correlate to observed y-type fragments, purple letters indicate sites where both b- and y-type ions were observed. These data strongly support the bilin bridging residues 50 and 61. These data suggest a bilin crosslinking Cysteine 50 to Cysteine 61. See Supplementary Table 4 for list of matched fragments.

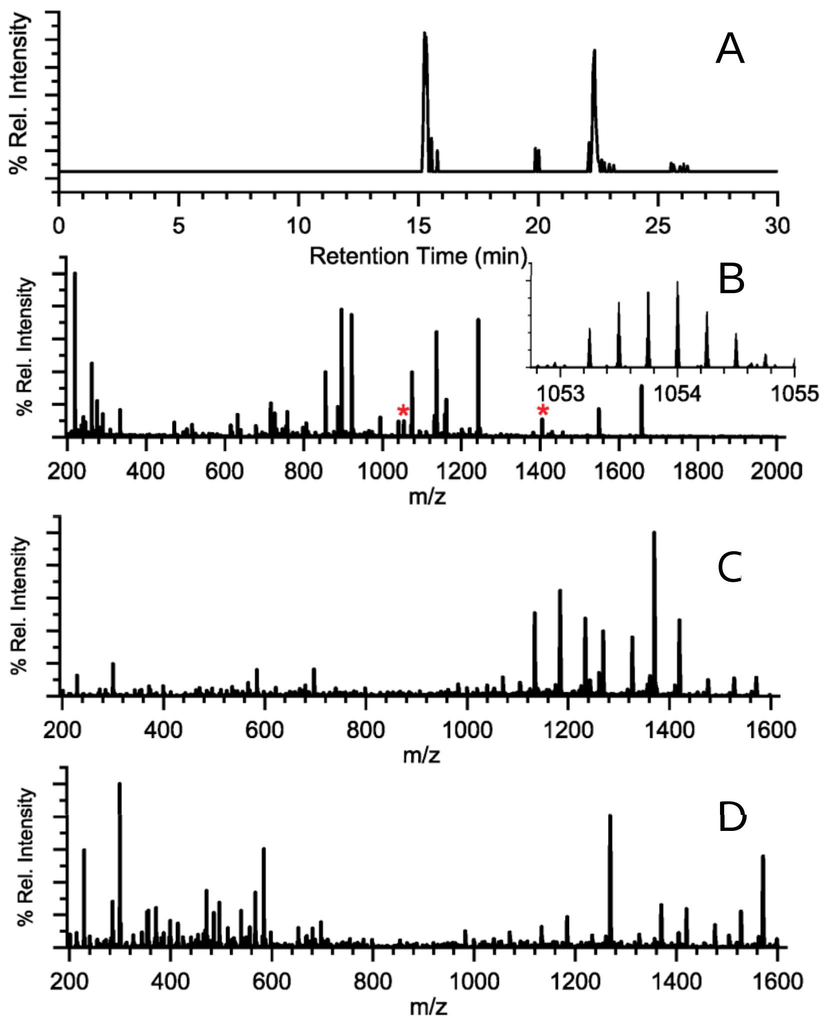

Supplementary Figure 7: Representative extracted ion chromatogram, precursor mass spectrum, and tandem mass spectra for peptide 38-78 with no bilin and a disulfide bridging positions 61 and 73 from WHCpeB(H141L)A+ RSCpeZ/CpeS+ RSMpeV co-expression. A: Extracted Ion Chromatogram for precursor ion at m/z 1404.6674 (no bilin, 61/73 disulfide); peak apex is at 22.35 minutes. B. Mass Spectrum of the 22.35 minute peak, red stars indicate 38-78 peptide peaks. Inset shows $(M+4H)^{4+}$ isotopic cluster. C. Tandem mass spectrum of m/z 1053.8 @ 22.35 minutes. D. Tandem mass spectrum of m/z 1404.7 @ 22.35 minutes. See Supplementary Table 5 for list of matched fragments. Note: peak with mass 1404.66 at 15.2 minutes in A gave entirely different tandem mass spectra and different monoisotopic peaks.

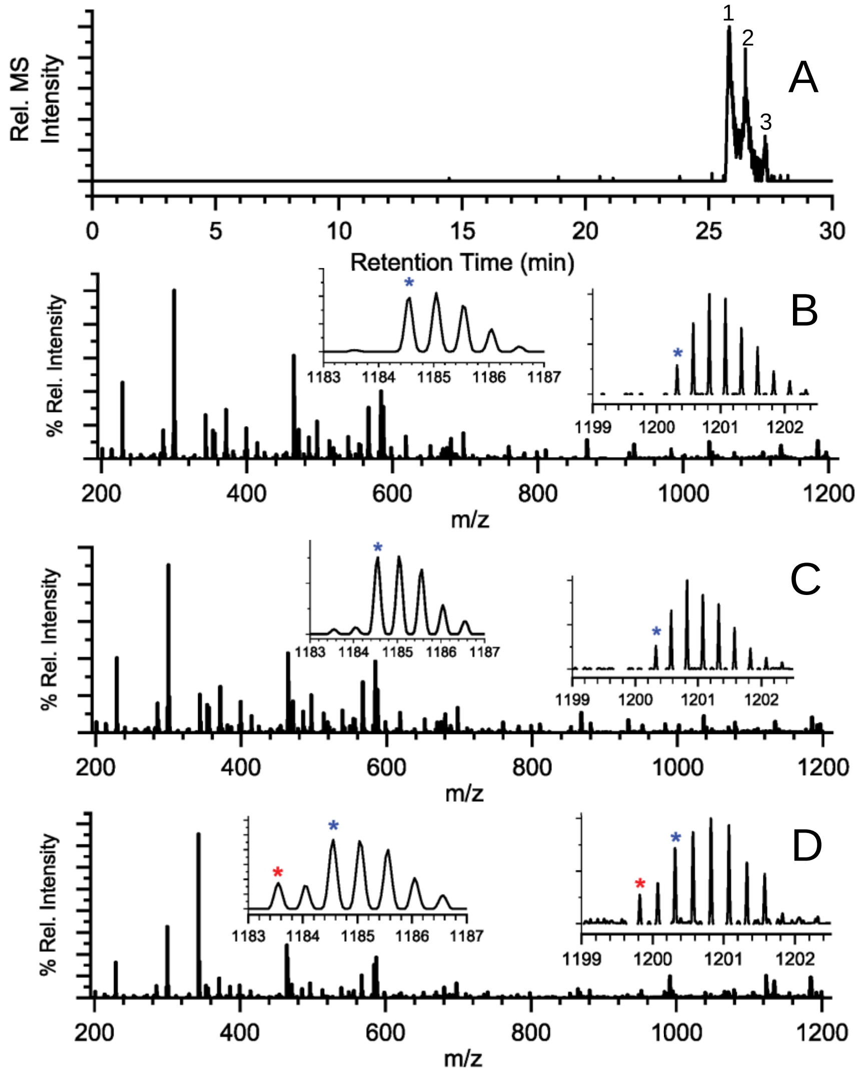

Supplementary Figure 8: Representative extracted ion chromatogram, precursor mass spectra, and tandem mass spectra for three versions of peptide 38-78 with a singly attached bilin at position 50 from WHCpeB(H141L)A+ RSCpeZ/CpeS+ WHMpeV co-expression. A. Extracted Ion Chromatogram for precursor ion at m/z 1200.8254; peak apexes are at (1) 25.83, (2) 26.47, and (3) 27.28 minutes. B. Tandem mass spectrum of 1200.8 at 25.83 minutes. Right inset shows $(M+4H)^{4+}$ cluster; left inset shows area around y_{22}^{2+} fragment ion. See Table S6 for list of matched MS-MS peaks. C. Tandem mass spectrum of m/z 1200.8 @ 26.47 minutes. Right inset shows $(M+4H)^{4+}$ cluster; left inset shows area around y_{22}^{2+} fragment ion. D. Tandem mass spectrum of m/z 1200.8 @ 27.28 minutes. Right inset shows $(M+4H)^{4+}$ cluster; left inset shows area around y_{22}^{2+} fragment ion. See Supplementary Tables 6 and 7 for a list of matched MS-MS peaks. Blue stars indicate monositopic peaks for a bilin at Cysteine 50 with free thiols at Cysteines 61 and 73. Red stars indicate monoisotopic peaks for a bilin at Cysteine 50 with a disulfide bond between Cysteines 61 and 73.

References:

Carrigee, L., Mahmoud, R.M., Sanfilippo, J.E., Frick, J.P., Strnat, J.A., Karty, J.A., et al. (2020a). CpeY is a phycoerythrobilin lyase for cysteine 82 of the phycoerythrin I α-subunit in marine *Synechococcus*. *BBA Bioenergetics* 1861. doi: 10.1016/j.bbabio.2020.148215.

Carrigee, L.A., Frick, J.P., Karty, J.A., Garczarek, L., Partensky, F., and Schluchter, W.M. (2020b). MpeV is a lyase isomerase that ligates a doubly linked phycourobilin to the β-subunit of phycoerythrin I and II in marine *Synechococcus*. *J . Biol . Chem.* 296(100031)**,** 1-13. doi: 101074/jbc.RA120.015289.

Dammeyer, T., Homann, E., and Frankenberg-Dinkel, N. (2008). Phycoerythrobilin Synthase (PebS) of a Marine Virus: Crystal Structures of the Biliverdin Complex and the substrate-free form. *J Biol Chem* 283(41)**,** 27547-27554.

Garczarek, L., Guyet, U., Doré, H., Farrant, G.K., Hoebeke, M., Brillet-Guéguen, L., et al. (2021). Cyanorak v2.1: a scalable information system dedicated to the visualization and expert curation of marine and brackish picocyanobacteria genomes. *Nucleic Acids Res* 49(D1)**,** D667-D676. doi: 10.1093/nar/gkaa958.

Humily, F., Partensky, F., Six, C., Farrant, G.K., Ratin, M., Marie, D., et al. (2013). A gene island with two possible confirurations is involved in chromatic acclimation in Marine *Synechococcus*. *PLOS One* 8(12)**,** e84459.

Hussain, H., and Chong, N.F.-M. (2016). Combined Overlap Extension PCR Method for Improved Site Directed Mutagenesis. *Hindawi Publishing Corporation BioMed Research International* 2016**,** 1-7.

Kronfel, C.M., Hernandez, C.V., Frick, J.P., Hernandez, L.S., Gutu, A., Karty, J.A., et al. (2019). CpeF is the bilin lyase that ligates the doubly linked phycoerythrobilin on β–phycoerythrin in the cyanobacterium *Fremyella diplosiphon*. *J. Biol. Chem.* 294(11)**,** 3987-3999.

Léger, C., Meo, T.D., MagaliAumont-Nicaise, ChristopheVelours, Durand, D., Sierra-Gallay, I.L.d.l., et al. (2019). Ligand-induced conformational switch in an artifcial bidomain protein scafold. *scientific reports* 9(1178). doi: 10.1038/s41598-018-37256-5.

Ritter, S., Hiller, R.G., Wrench, P., Welte, W., and Diederichs, K. (1999). Crystal structure of a phycourobilin-containing phycoerythrin at 1.9 Å resolution. *J. of Struct. Biol.* 126**,** 86-97.

Sanfilippo, J.E., Garczarek, L., Partensky, F., and Kehoe, D.M. (2019). Chromatic Acclimation in Cyanobacteria: A Diverse and Widespread Process for Optimizing Photosynthesis. *Ann. Rev. Microbiol.* 73**,** 407-433. doi: 10.1146.

Sanfilippo, J.E., Nguyen, A.A., Karty, J.A., Shukla, A., Schluchter, W.M., Garczarek, L., et al. (2016). Self-regulating genomic island encoding tandem regulators confers chromatic acclimation to marine *Synechococcus*. *Proc. Natl. Acad. Sci. USA* 113(21)**,** 6077-6082. doi: 201600625.

Shukla, A., Biswas, A., Blot, N., Partensky, F., Karty, J.A., Hammad, L.A., et al. (2012). Phycoerythrin-specific biln lyase-isomerase controls blue-green chromatic acclimation in marine *Synechococcus*. *Proc. Natl. Acad. Sci. U. S. A.* 109(49)**,** 20136-20141. doi: 10.1073.

Six, C., Thomas, J.-C., Garczarek, L., Ostrowski, M., Dufresne, A., Blot, N., et al. (2007). Diversity and evolution of phycobilisomes in marine *Synechococcus* spp.: a comparative genomics study. *Genome Biol.* 8(12)**,** R259.

Wilbanks, S.M., and Glazer, A.N. (1993). Rod structure of a phycoerythrin II-containing phycobilisome I: organization and sequence of the gene cluster encoding the major phycobiliprotein rod components in the genome of marine *Synechococcus* sp. WH8020. *J. Biol. Chem.* 268**,** 1226-1235.
